# Recent advances of Au@Ag core–shell SERS‐based biosensors

**DOI:** 10.1002/EXP.20220072

**Published:** 2023-02-07

**Authors:** Gul Awiaz, Jie Lin, Aiguo Wu

**Affiliations:** ^1^ Cixi Institute of Biomedical Engineering, International Cooperation Base of Biomedical Materials Technology and Application, Chinese Academy of Sciences (CAS) Key Laboratory of Magnetic Materials and Devices and Zhejiang Engineering Research Center for Biomedical Materials Ningbo Institute of Materials Technology and Engineering, CAS Ningbo China; ^2^ University of Chinese Academy of Sciences Beijing China; ^3^ Advanced Energy Science and Technology Guangdong Laboratory Huizhou China

**Keywords:** Au@Ag core–shell nanoparticles, biosensors, SERS probes

## Abstract

The methodological advancements in surface‐enhanced Raman scattering (SERS) technique with nanoscale materials based on noble metals, Au, Ag, and their bimetallic alloy Au–Ag, has enabled the highly efficient sensing of chemical and biological molecules at very low concentration values. By employing the innovative various type of Au, Ag nanoparticles and especially, high efficiency Au@Ag alloy nanomaterials as substrate in SERS based biosensors have revolutionized the detection of biological components including; proteins, antigens antibodies complex, circulating tumor cells, DNA, and RNA (miRNA), etc. This review is about SERS‐based Au/Ag bimetallic biosensors and their Raman enhanced activity by focusing on different factors related to them. The emphasis of this research is to describe the recent developments in this field and conceptual advancements behind them. Furthermore, in this article we apex the understanding of impact by variation in basic features like effects of size, shape varying lengths, thickness of core–shell and their influence of large‐scale magnitude and morphology. Moreover, the detailed information about recent biological applications based on these core–shell noble metals, importantly detection of receptor binding domain (RBD) protein of COVID‐19 is provided.

## Graphical abstract

Bimetallic alloy Au–Ag surface‐enhanced Raman scattering (SERS) biosensors exhibit ultrahigh SERS sensitivity. Au–Ag SERS‐based biosensors have been utilized in biological components detection, including; proteins, antigens antibodies complex, circulating tumor cells, DNA, and RNA (miRNA), etc.

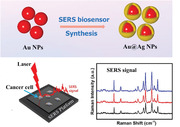

## 1 INTRODUCTION

Surface‐enhanced Raman spectroscopy (SERS) is a significant methodology of spectroscopy that permits the exceptionally subtle systemic sensing of analytics. It is one of the best technique to identify the substances, recognizing chemicals and molecular structures.^[^ ^1^ ^]^ By amplifying the value of scattering signal coming from the interaction among noble metal target and excitation beam known SERS, it can increase the sensitivity for cellular detection,^[^ ^2^ ^]^ viral identification,^[^ ^3^ ^]^ and biomolecular detection.^[^ ^4^ ^]^ This technique can be further utilized in the bioimaging, environmental studying, and other biomedicine tests.^[^ ^5^ ^]^

It has already been a known factor that gives insight into the structural, quantitative, and qualitative details of analytes through the identification of the vibrational bands of the functional groups that can be provided by Raman spectroscopy. Although in previous studies, this technique was typically not considered to be sensitive, and in most of the occurrence, it required an expanded integration time resulting in potential destruction of the sample.^[^ ^6^ ^]^ From the time of discovery in 1928 to the 1960s, Raman measurements were constrained by the purity of the solvents. So, the enhancement was done in the domain of analyte concentration and attainable analytes following the development of the laser in the 1960s, but frail signals still restricted the profitability of this case for chemical examination. Nonetheless, the circumstances changed since 1970s enhanced Raman scattering of molecules were detected by utilizing a roughened metal surface like Ag, which improved by a few orders of magnitude for the Raman signal of an analyte situated in its accessibility.^[^ ^7^ ^]^ In 1974, Fleischmann and colleagues accidentally observed the surface area enhancement effect while they were measuring the Raman scattering on rough silver electrodes.^[^ ^8^ ^]^ Jeanmarie and Van Duyne projected an enhancement factor of 10^5^–10^6^,^[^ ^9^ ^]^ while Albrecht and Creighton put forward a theory of resonant Raman involving plasmon excitation.^[^ ^10^ ^]^ Moskovits found a connection between the enhanced fields and SERS values which were caused by localized surface plasmons in metals bearing nanostructures.^[^ ^1^ ^]^ Moreover, Philpott presented the idea of connection with plasmon excitation.^[^ ^11^ ^]^ After some time with many subsequent experiments, it was validated that Raman scattering signals of analytes can be enhanced by the use of rough surfaced noble metal films or nanoscale patterns. These enhancements can go up to a factor of 10^4^–10^8^.^[^ ^12^ ^]^ Subsequently, scientists have created magnificent advancements regarding sufficient apprehension of the basic SERS ideology.

It caught the attention of researchers through the enrichment in productivity of electromagnetic fields produced by the agitation of confined surface plasmons.^[^ ^13^ ^]^ It is a procedure of hypersensitive detection, which could be utilized in molecular measurements for detailed descriptions. SERS spectrum has prominent characteristics of high sensitivity, adequate discrimination, anti‐interference, no light bleaching, and swift detection.^[^ ^14^ ^]^ SERS spectrum has the advantages that it does not require an improvement step, and the discernment procedure is very convenient to handle in it.^[^ ^15^ ^]^ As samples in small amounts can be handled correspondingly, directing to briefly average observation time (few minutes) for each trial, a comparatively transferable Raman spectrometer makes this mechanization facile to Publicize, and so forth.^[^ ^16^ ^]^ Therefore, SERS spectrum contains vast range of applications, including biomedical diagnosis^[^ ^17^ ^]^ and environmental analysis.^[^ ^18^ ^]^ SERS contains two techniques which are known as intrinsic and extrinsic SERS.^[^ ^19^ ^]^ Intrinsic SERS basically focuses on the dimensional analysis of the marked molecule and in the result; exclusive spectra of the molecular vibrational moment are attained and denoted as a Raman signature. The term, extrinsic SERS, basically explains the phenomenon of the analysis and calculation of targeted molecule due to the attained spectrum belonging to a Raman reporter instead of the marked molecule. The use of Raman reporters in SERS is essential to be close or adsorbed on the exterior of SERS‐active substrates as the dependency of electromagnetic intensification is chiefly dependent on the linking extent of the reporters and substrate.^[^ ^19^, ^20^ ^]^

The contraption following SERS improvement is consistently a realm of interest for number of research to recognize and describe the occurrence. Normally, the enhancement mechanisms for SERS involve two different types of mechanisms: chemical enhancements and electromagnetic enhancements.^[^ ^21^ ^]^ Chemical enhancements (CM) have limited effect on Raman enhancement typically around 10–10^2^ times. CM enhancements are usually caused by exchange of electrons between the metal and molecule, and are contributed by three different processes including; (a) enhancement due to ground state chemical interactions; (b) resonance Raman enhancement; (c) charge‐transfer. CM enhancement is short‐range effect, as the distance between sample and substrate is in angstroms.^[^ ^22^ ^]^ Plasmon resonance enhancement processes commonly referred to as electromagnetic enhancement (EM) have major effect on Raman enhancement that is usually about 10^13^–10^15^ times. EM enhancement arises due to light amplification governed by local surface plasmon resonance (LSPR). LSPR from substrate is excited by the far‐field incident light, and focus light on tip, edge, or gap on nanoscale, consequently increasing the electromagnetic intensity by 2–5 folds. Local oscillators, like dipole or quadruple, can also excite the LSPR and are reradiated into the far‐field.^[^ ^23^ ^]^ Here, specimens are commonly settled on or at contiguity of nanostructured metallic substrate, called SERS substrate where the improvement transpires as the consequence of reciprocity amidst the approaching light, metallic surface, and the selected sample molecule.^[^ ^24^ ^]^

### 1.1 LSPR

LSPR is a concept dealing with noble metallic elements for their nano‐scale structures and spectroscopic analysis relating to photothermal improvements. It is considered very beneficial in SERS‐based studies and research related to it. In this methodology, the rugged uneven surfaces are changed to surface plasmons, and they show sensitivity to projected laser‐beams as it affects the outer shell electrons of metals. This concept is formulated as the rate of projected light‐beam reaches near the rotating frequency of the surface plasmon.^[^ ^25^ ^]^ The elevated Raman scattering is obtained by establishing very intense local electric fields on surface sites. As in reference to this concept, EM (electromagnetic) domain is evidently expanded with the excitation of main central core of metallic nanoparticles, the LSPRs of the branch angle, and the LSPRs hybridized between the central nucleus and the branch angle. It gives rise to impactful SERS results. The conformational regulation is essential in maintenance and authorized dominance of structural framework.^[^ ^26^ ^]^ The role and efficiency of noble metals especially gold and silver‐based NPs make it a very impactful point in LSPR based works.^[^ ^27^ ^]^

It has been described in earlier works that LSPR spectral values of gold nanoparticles are closely related to structural regulation and extent of distribution, and they have tendency to adjust according to variating number of NPs and intended outcome.^[^ ^28^ ^]^ The applications like biosensing with nanoscale analysis capability are the beneficial gain from the effective and delicate SNR (signal‐to‐noise‐ratio) performance.^[^ ^29^ ^]^ Moreover, the fabrication of noble metal‐based nanoparticles and their structural framework upgradation have the tendency to impact values of this localized resonance. The major conceptual points in this structural upgradation will be the excessive development of nanoparticles and etching.^[^ ^30^ ^]^

Surface Plasmon Resonance can be observed on various noble metal surfaces, including gold and silver. Noble metal substrates usually have a large surface area and a strong attraction for noble metal nanoparticles. Therefore, to further maximize their potential, a novel class of Au/Ag bimetallic biosensors has been created using these Au and Ag nanoparticles to detect distinct analytes in varying media. These Au/Ag bimetallic biosensors demonstrate a unique SPR effect due to generating a higher signal when the sensor is exposed to Au‐Au, and Ag‐Ag complexes in the near‐infrared region (NIR).^[^ ^31^ ^]^ This individual SPR effect results from the Ag‐NPs binding to the Au NPs, which in turn causes a change in the optical properties of the Ag‐NPs, including a shift in the optimal wavelength at which the SPR effect is detected, increasing the SPR signal.^[^ ^32^ ^]^ Using a range of Au/Ag bimetallic NP concentrations allowed researchers to illustrate the inherent capability of the SPR effect to improve the detection capabilities and sensitivity of biosensors.

#### 1.1.1 Hotspots

“Hotspots” are SERS‐active structures present in the NP gaps, in roughened metal surface, and in other sharp features of noble and coinage metals (Au, Ag, and Cu). The electromagnetic field is localized in these hotspots which seem like unevenly distributed nano‐gaps between the particles, and between the particles and substrates.^[^ ^33^ ^]^ These strongly enhanced local electromagnetic field regions are divided in to three main generations of developments (a) when similar types of nanoparticles for example, nanospheres are organized in a homogeneous mixture, single nanostructure hotspots are formed with moderate SERS activity known as first generation hotspots.^[^ ^34^ ^]^ (b) Coupled nanostructures having controlled nanogaps generate second generation SERS hotspots, these hotspots govern excellent SERS activity and can even perform single molecule detection. But sometimes, these generation hotspots are unsuitable because some materials like silicon cannot penetrate into nanogaps.^[^ ^34^, ^35^ ^]^ (c) Hybrid structures consisting of plasmon nanostructures and probe materials are third generation SERS hotspots that can directly recognize hotspots on the surface of materials. While designing the material of complex nanostructure, the third generation hot spots can be shifted to the surface of material to be measured as the LSPR, and local electric field is influenced by dielectric properties of materials.^[^ ^36^ ^]^ EM enhancement depends upon the roughness of substrate. EM is long‐range effect as the sample is placed 1–10 nm apart from the substrate.

The LSPR has the tendency to precisely analyze and identify multiple chemical and biological molecular components while relying heavily upon the molecular framework and structural efficacy and upgradation level of nanoparticles.^[^ ^37^ ^]^ The formulated nanoscale area of “hot spot” contains very efficient and robust surface EM field and it further amplifies the Raman performance and sensing. This Raman working and performance also get impacted by the adsorption of Raman molecules on the hot spots nanomaterials. The importance of limited number of hot spots is also evident by the factor that desired molecule is needed to reach and stay in it for proper discernment. The nonspecific placements of Raman molecules on the SERS substrate give rise to the terrible replication of its signals.^[^ ^38^ ^]^

### 1.2 SERS nanoparticles

Semiconductors and noble metals are in race of exploring novel SERS substrate for effective biosensing. For example, semiconductors‐based substrates have good biocompatibility and reproducibility in fingerprint spectral detection.^[^ ^39^ ^]^ There are various other semiconductor based SERS substrates in use like TiO_2_ nanosheets,^[^ ^40^ ^]^ ZnO nanoparticles ZnO nanosheet.^[^ ^41^ ^]^ and Cu_2_O nanoparticles.^[^ ^42^ ^]^ After the detailed studies on the improvements of their structures, TiO_2_ nanowires^[^ ^43^ ^]^ and Ta_2_O_5_ nanorods^[^ ^43^ ^]^ have served as SERS substrates. The efficient maneuvering techniques like element doping,^[^ ^41a^ ^]^ surface defects stats,^[^ ^44^ ^]^ and efficient interfacial charge transfer in the amorphous semiconductor molecules lead to the attainment of more effective SERS semiconductors.^[^ ^39^ ^]^ Anyhow, practical applications of semiconductors especially in biosensing have been limited owing to their weak enhancement factor EFs.^[^ ^45^ ^]^

SERS is used for plasmonic nanomaterials to increase the low Raman signals by using the optical and chemical properties. To control the Raman signal, we can increase the Raman scattering cross‐section if we put it in the vicinity of plasmonic NPs, this is all because of the localized EM resultant of a localized surface plasmonic resonance (LSPR). A combination of a long‐range electromagnetic (EM) and short‐range chemical enhancement mechanism can bring the enhancement. LSPR can cause EM which in turn contributes a lot to the enhancement mechanism of the SERS. An amplified local electromagnetic field is a result of a light that falls near or on the surface of metallic nanoparticles (NPs).^[^ ^46^ ^]^ We can have strong SERS signals through utilizing the metal nanostructure and SERS materials. To improve the SERS activity, SERS probes are produced and utilized in different sizes, shapes like nanospheres, multi branched NPs, etc.^[^ ^47^ ^]^ Single NPs could not give enough sensitive SERS signals, however, bimetallic Au and Ag NPs got a lot of attention in SERS because the bimetallic alloys such as Au–Ag are able to boosts the EM.^[^ ^5a^ ^]^ Among the noble metals, Ag and Au have the nanostructures as the typical examples of SERS substrates. Metallic nanoparticles manifest non‐identical electrical and optical properties in comparison to their volume substance; therefore, studies are highly invested in these nanoparticulate materials due to their applications in biosensing, optics, plasmonic, and biomedicine, amidst others.^[^ ^48^ ^]^ Bimetallic materials also have many advantages over the single NPs like they have dynamic electronic properties and they can enhance the signals of SERS board spectra of light.^[^ ^49^ ^]^ Moreover, Au–Ag combination can bring the exceptional enhancement property from Ag and biocompatibility and long‐term stability of Au nanostructures.^[^ ^50^ ^]^

A lot of unseen paths can be explored in the chemistry, biomedicine, and life sciences by molecular cognition and SERS‐based signal detection.^[^ ^46a^ ^]^ It also demonstrates a huge number of benefits: (i) sample preparation is easy,^[^ ^51^ ^]^ (ii) single molecule detection,^[^ ^52^ ^]^ (iii) molecular detection at ultra‐low concentrations, (iv) high throughput, (v) high selectivity due to possible unique fingerprint signature of analyte, (vi) no signal interference from water‐based analyte medium (vii) single laser beam with multiplexed sensing potential.^[^ ^53^ ^]^

Here in this study, we will comprehensively review the introduction of SERS based biosensors, with a major focus on the recent developments, properties, and advantages of Au/Ag alloy‐based materials as SERS, followed by designing principles, nanofabrication methods, and enhancement mechanisms of Au/Ag‐based SERS biosensors. Furthermore, summarizes the various applications of Au/Ag alloy‐based SERS bio‐probes in biological detection within past few years. Finally, future of SERS biosensors regarding difficulties and opportunities will be discussed. This paper will have the potential to be used as a reference for the foundation of the Au–Ag SERS‐based biosensors and their further development.

## 2 NOBLE METALS AS SERS MATERIALS

In this topic we will discuss the properties of pure noble metals and their alloys, their advantages, and disadvantages as SERS biosensor.

### 2.1 Gold (Au) as SERS material

Among the noble metal nanoparticles, Au and Ag nanoparticles are frequently utilized as SERS substrates due to their elementary, convenient, and frugal decentralize creation, such as addition of reducing agents to aqueous solution of the metallic salts.^[^ ^54^ ^]^ In previous studies, the formation of gold nanoparticles was mainly focused on the biosensing applications, such as the detection of circulating tumor cell (CTCs) from human peripheral blood through SERS.^[^ ^55^ ^]^ Further enhancements in different varieties of AuNPs (Spheres, Stars, Rods) were utilized, and amidst these, it was depicted that gold nanostars were highly sensitive with 1 cell/ml of limit of detection (LOD).^[^ ^56^ ^]^ Gold (Au) nanoparticles have been utilized for decades as efficacious SERS substrates.^[^ ^57^ ^]^ Exploration of applications in SERS has been increased and employed on the large scale such as applications in imaging and sensing, but the pattern and optimization of systematic substrates are still in demand as well.^[^ ^58^ ^]^ Gold nanospheres were also manufactured and identified conforming to their zeta potential, basic size, and UV/visible absorption conducive for the reorganization and improvement of the SERS biosensing ability.^[^ ^59^ ^]^ Nowadays, the synthesis and development of gold particles are performed by the procedures given below.

1. Seed growth method^[^ ^60^ ^]^
2. One‐step synthesis method^[^ ^61^ ^]^
3. Electron beam etching method^[^ ^62^ ^]^

First method is comparatively more productive, simple, and quick than other two methods, and it is very impactful in modulation of plasmonic optical performance and structural patterning.^[^ ^63^ ^]^ One of the major characteristic factors of plasmonic NPs is their capability to harvest heat energy from the conversion of solar light rays which can be useful for therapeutic treatment by electromagnetic radiation for carcinogenic cases tumors removal.^[^ ^64^ ^]^

In previous works, the SERS activities were researched for Au at nanoscale by employing different cyanide and amine‐based compounds.^[^ ^65^ ^]^ However, in these works, gold nanoparticles were randomly chosen and studied in various compositions and dimensions. The major consideration in these selections was the research of particles with variating and uneven conformities. The specificity for preparation in case of shape and proportional measurements was also adopted for work on gold nanoscale materials by employing photochemical method with help of UV–vis radiations.^[^ ^66^ ^]^ The impact on the SERS profile was evident by the shape variation noted in nanoparticles, and uneven and rugged structured NPs exhibited the best improvements and performance for desired applications in biological field.^[^ ^67^ ^]^

### 2.2 Silver (Ag) as SERS material

Zhang et al.^[^ ^68^ ^]^ explained the work to deal with the drawback of weak signals detection from Au nanoparticles usage. The application of Ag nanoparticles and superparamagnetic iron oxide nanoparticles with the ramification of encapsulation and triangular silver nanoprisms,^[^ ^69^ ^]^ enhancement, ransom, and detection aided in the detection of CTCs through SERS.^[^ ^69^ ^]^ For research of SERS, Ag is the most effective substance due to its d‐s band gap which is in the UV domain, and it does not damp out the plasmon band like it firmly does for Au. Another factor for Ag being the most effective is due to the capability of Ag NPs to manifest much powerful surface electric field undergoing plasmonic excitation state than the corresponding Au NPs.^[^ ^70^ ^]^ Nonetheless, in the case of Ag NPs, their conduction in SERS abides the oxidation procedure as the charge transmission to the analyte molecule is obstructed by the oxidized Ag surface following the given statement of chemical enhancement mechanism. It is analytically conventional that most of the fierce signals in SERS are gained from molecules adsorbed to silver surfaces that are microscopically rutted, such as Ag NPs and Ag colloids of several configurations.^[^ ^71^ ^]^

The Ag NPs in SERS face issues of oxidative reactions.^[^ ^72^ ^]^ This problem impacts the electron movement to analytes. The low sustainability, low precipitation, and lower agglomeration are disadvantages of Ag NPs.^[^ ^73^ ^]^ Though, the robust oxidative reaction of Ag NPs discharges silver ions causes harmful consequences on the biological structures by prompting cytotoxicity, genotoxicity, and immunological responses, which often leads to cell‐death.^[^ ^74^ ^]^ The application of these nanoparticles contains a lot of unsought arbitrary issues related to bio‐systems. They potentially pose a serious threat of harming blood and brain system of living subjects especially mankind in case of large‐scale usage. The potential of these particles to diffuse through transcellular transport of capillary endothelial cells and probably toward further dangerous parts could be fatal. In one other reported work, silver can potentially make active silver ions in drug suspension for therapeutic reasons, but it has direct serious side‐effects on biological systems of patients.^[^ ^75^ ^]^ The elevations of ionic silver on earth's surface and its aquatic resources have reached up to 22.7 ppm and 0.76 ppm, respectively, which is believed to be more poisonousness than its elemental and nanoscale forms.^[^ ^76^ ^]^

### 2.3 Silver (Ag) and gold (Au) as alloys

Most researches in SERS have been executed by the employment of Ag and Au nanostructures. Indeed, the enhanced Raman signals can be gained from Ag nanoparticles, but they are less likely to be considered for biological detections due to their toxicity. On the contrary, Au nanostructures are favored on a large scale, but they exhibit only basic SERS effects. Consequently, it would be right to link Au/Ag as bimetallic method such as core/shell or alloy to fully explore their potentials of physicochemical properties/potential through SERS research to investigate biomolecules.^[^ ^77^ ^]^ Consequently, as mentioned above, Ag NPs are not favored like Au NPs due to their proportionately adequate plasmonic characteristic in contrast to Au NPs. Accordingly, enviable appropriate bimetallic system is pivotal that conditioning upon their applications for which the system is deliberated. From the previous studies, it has been implicated those structures like Ag–Au alloy may root inauspicious repercussions when it initiates interconnections with the biological systems as Ag subsists on the surface of alloy particles. On the contrary, in core/shell like formation, it is feasible to regulate which metal (either Ag or Au) should interrelate with biological system by creating congruous Ag–Au standard or transposing core/shell structures. By altering the depth of core/shell layer, it is possible to tune their plasmonic characteristics on large scale. The plasmonic reaction of the system is significant as it governs which domain of wavelength, either visible or infra‐red (IR) or ultraviolet (UV) should be utilized as an exploring wavelength in the research of biological systems.^[^ ^77^, ^78^ ^]^ For general biological research, it is more doable to utilize visible light and IR wavelength in the occurrence of researching tissues.

The individual flaws of gold and silver nanostructures can be avoided via the hybridization process, and significant research efforts have been done toward the rational designing of bimetallic Ag–Au materials in a core–shell fashion. Although gold nanostructures have high stability and monodispersity, their LSPR property is significantly less than that of silver nanostructures. Unfortunately, Ag with strong plasmonic properties has poor resistance to oxidation and is easily aggregated, which limits its practical value. Consequently, it is crucial to combine the high stability of gold with the unique plasmonic properties of silver in a single nanostructure. The chemical stability and robust optically responsive properties of Ag–Au Nanostructures, like the position and bandwidth of SPR peaks, were discovered to be substantially reliant on the specific proportions of the metals in the composite alloy structure.^[^ ^79^ ^]^

Au/Ag alloys are a material that can have high spin‐electron relaxation properties at room temperature. Therefore, the Au/Ag alloy‐based SERS biosensing is the most appropriate platform for applications requiring room‐temperature spin‐electron relaxation. Moreover, The SERS response of Au/Ag alloys was found to be the highest at low frequencies and the lowest at high frequencies.^[^ ^80^ ^]^ Because the electrons in the Au/Ag alloy are localized at low frequencies, they are thus sensitive to the magnetic field. At high frequencies, electrons can be delocalized, allowing them to pass through the magnetic field unaffected. It makes Au/Ag alloys the optimum platform for high low‐frequency performance and low high‐frequency performance.^[^ ^81^ ^]^

The Au/Ag alloys possess low density and allow for a significant increase in material sensitivity.^[^ ^82^ ^]^ As a result, the background noise is reduced, and the signal‐to‐noise ratio is improved. The Au/Ag alloys also allow for higher frequency response, minimizing the background noise of the material. These alloys also allow for a smaller footprint, resulting in a material that uses less power. In addition to that, high electrical conductivity has been demonstrated in Au/Ag alloys, making them the most suitable material for usage in SERS systems. The exceptional electrical conductivity of the alloys is due to the significant volume fraction of silver which is an excellent conductor of electricity. Furthermore, the key advantages of Au/Ag alloys are their chemical and thermal stability and their improved mechanical qualities.^[^ ^83^ ^]^ They also generate a greater SERS signal than pure gold and silver platforms.

As the researchers around the globe are putting more thought process and efforts for the synthesis of various morphologies of plasmonic noble metals that is, various shapes and size resulting in better optical properties and ultimately enhancing SERS behaviors, there is an increasing demand for the new synthetic methodologies like solution based chemical methods, etc.^[^ ^84^ ^]^ The solution based chemical method has been proven to be the best among all for obtaining a particular size of nanoparticles because it can easily be supervised from nucleation and at each growth step as well, but these solutions surely contain uncertain chemicals which effects the toxicity, making it unfit for biosensing.^[^ ^85^ ^]^ So to synthesize biocompatible metallic NPs, natural reducing agents like sodium citrate have been used as aqueous synthesis methods instead of solution based chemicals.^[^ ^86^ ^]^ Although, aqueous synthesis has been widely used to prepare biocompatible NPs which have been applicable in biosensing, the quest of finding more effective way to synthesize NPs led to green synthesis of metallic NPs by using plant extracts as reducing agents.^[^ ^87^ ^]^ The use of these naturally extracted reducing agents helps to attain highly biocompatible and nontoxic materials.^[^ ^88^ ^]^ Starting from Au NPs to the preparation from extract of *Acorus calamus* rhizome,^[^ ^89^ ^]^ extracts of *Elaeis guineensis* leaves^[^ ^90^ ^]^ serving as reducing agents and Ag NPs prepared from leaves extractions of various plants like *Phyllanthus urinaria*, *Pouzolzia zeylanica*, and *Scoparia dulcis* leads to preparation of bimetallic NPs.^[^ ^91^ ^]^Various studies for the green synthesis of Au–Ag bimetallic materials have been reported. Gopinath et al. successfully synthesized core@shell Au–Ag NPs with an average 20 nm from the extract of *Gloriosa superba*.^[^ ^92^ ^]^ In another study, extract of *Azadirachta indica* has been used for preparation of Au–Ag bimetallic core@shell NPs^[^ ^93^ ^]^ as well as fenugreek, coriander, soybean, and clove buds extracts serving as reducing agent for preparation of Au–Ag bimetallic materials.^[^ ^94^ ^]^ Recently, PE of *Pulicaria undulata* (L.) was employed to synthesize Au–Ag bimetallic NPs, whereas effects of concentration of PE were also observed. Interestingly, Photochemical constituents in extract of *P. undulata* stabilized the Au–Ag bimetallic NPs because it was not only working as reductants but also serving as capping agent.^[^ ^95^ ^]^ In green synthesis, not only plants but microorganisms also put their role in formation of ecofriendly, sustainable, energy efficient, and biocompatible biosynthesized bimetallic NPs.^[^ ^96^ ^]^ The biomolecules present in microorganisms like proteins deoxyribonucleic acid (DNA) biconjugated with NPs serve as capping agents and enhance bioactive properties that is, protein extract of *Deinococcus radiodurans* microorganism used to synthesize Au NPs, Ag NPs, and Au–Ag bimetallic NPs.^[^ ^96^, ^97^ ^]^

## 3 SERS ENHANCEMENT AND VARIOUS FACTORS EFFECTING TO ACHIEVE HIGH SERS INTENSITY

Au and Ag are the two most frequently used SERS substrate due to their tunable LSPR bands in vis‐NIR domain, which are the most suitable for resonant agitation of the nanoparticle‐analyte composites. However, from the earlier research, it has been seen that, Ag NPs generally give excessive SERS enhancement element than the corresponding AuNPs.^[^ ^98^ ^]^ While Au NPs discover various applications as SERS compared to Ag NPs. The durable firmness against physical and chemical oxidation along with accompanying improved biocompatibility due to lower toxicity make Au NPs more flexible and ordered in comparison to silver Ag NPs which endure from their small firmness to build up clusters and manifest toxicity due to the generation of in situ Ag^+^. As an outcome of the appropriate characteristic features of gold (Au) nanoparticles (NPs), Au NPs are found more acceptable in vivo applications compared to their silver (Ag) counterparts. Nonetheless, Au and Ag core–shell NPs have attained a functional spot in the segment of SERS active substrate, as they do not only supply elevated SERS enhancement regulation like Ag NPs but also manifest high firmness and homogeneity such as Au.^[^ ^99^ ^]^

The induced EM field is produced at the core by interaction with light and in consequence, core–shell of Au and Ag NPs manifest Ag like optical belongings as its outer shell of NPs which are created from silver (Ag). Therefore, their LSPR band can be tuned in consequence of the SERS occupation by replacing the comparative size between shell and core.^[^ ^100^ ^]^ This can be attained by replacing the size and structure of the core or by replacing the shell breadth, and therefore, Au and Ag NPs have become preferable SERS active substrate due to these factors as compared to nanoparticles comprising of single substance either Ag or Au.

### 3.1 LSPR and thickness

When two metals with dissimilar dielectric constants are put down besides each other in a core arrangement, an LSPR shift will take place. By utilizing the Mie theory, researchers have forecasted that enlarging the shell denseness of a nanoparticle transferred the LSPR from high to low wavelengths. This transfer was validated analytically in most of the cases. For instance, Oldenburg et al., noticed a blue shift in the extinction peak as the gold shell denseness enlarged over a silica core, once the shell was completely established. In the occurrence of silver‐gold core–shell ordering, there was a red shift as gold comprised of a plasmon resonance peak at a lengthy wavelength as compared to silver. As the gold shell denseness expanded, the absorbance peak also decreased. Additionally, in these substitutions, gold–silver or silver–gold core–shell ordering formed two plasmon resonance peaks equivalent to gold and silver. It was found out the generally, the substitutions in LSPR wavelengths could be amended utilizing core–shell nanoparticles by operating constitution substances and shell denseness amidst other parameters.^[^ ^101^ ^]^

In a research, Au/Ag core/shell and inverted Ag/Au core/shell were synthesized and examined for the best SERS effective response to for the detection of methylene blue (MB and methylene orange MO.^[^ ^102^ ^]^ It was found out that though Ag/Au core/shell nanoparticles displayed clearly the general peaks of probes, the enhancement and capability to detect probes was far better for the Au/Ag core shell nanoparticles.

### 3.2 Wavelength dependence effect

Optimized wavelength for SERS activity is also an important factor for achieving better SERS sensitivity. It was explained in a study that if the excitation and scattering wavelengths sat at two sides of extinction peak of SPR, it would help to obtain high EFs SER spectra. A good excitation wavelength has a characteristic that it must be shorter than LSPR, and the displacement between them is ideally half of vibrational frequency. Therefore, as an excitation laser shorter wavelength laser is preferable, the Raman intensity is proportional to fourth power of incident frequency but in case of noble metals like Au and Ag nanostructures, SERS effects could not take place in shorter wavelength due to having interband transitions that transformed incoming optical fields in to heat rather than transforming in to local field enhancement. Therefore, the most suitable point is to select the excitation laser according to the molecular absorbance of SERS substrate which would also help to reduce the interference of fluorescence background.^[^ ^103^ ^]^

### 3.3 Distance

The relationship between the local electromagnetic mechanism EM and the SERS intensity I have been deliberated to be I ∝ |E|^4^ and the relationship of E and the distance between analytes and nanoparticles, D, have been explained as E ∝ (1/D) ^12^. From these two equations, it is perceived that a minute reduction of distance consequences in a magnificent enhancement of intensity. It was found that vast field strength could only be manufactured by the molecules which were on the metal surface or near the metal surface. Therefore, the best way to achieve successful detection of SERS would be to create the contact between analyte molecules with SERS substrate surface as many points and as near as feasible.^[^ ^104^ ^]^

### 3.4 Effects of size, shape, and shell thickness

In modern scientific and commercial fraternities, noble metal nanoparticles are becoming more valuable for their contribution in multiple sectors like SERS process upgradation, elevated quality of catalysis in synthetic projects, sustainable pharmaceutical diagnostic and medicinal products, optics applications, biosensing, photovoltaics, chemo‐photothermal therapy, etc.^[^ ^104^, ^105^ ^]^ In comparative analysis, bimetallic core–shell nanoparticles have been found to be more efficient and impactful in technological and industrial sectors with their superior properties than pure metal‐based NPs. Based on the characteristic features like size, shape, and composition, nanoparticles get affected by great deal of the quality of them. In the case of core–shell, the surface plasmon resonance (SPR) of the nanoparticles was learned to be reliant greatly on the above explained basic features, however, each constituent does have its impact and role in shaping overall nature of it.^[^ ^106^ ^]^ The two metals of Gold and Silver from group of precious noble metals have traditionally been in spotlight of commercial as well as scientific community due to their advanced synthetic techniques, extensively adaptive plasmonic wavelengths, and excessive amount of absorption/scattering cross‐sections, and polarization‐subtle plasmonic approaches.

Due to extremely effective performance of SERS for morphological characteristics of NPs, the development of novel metallic nanoscale materials has increased with focus on their basic features with respect to their role as SERS active substrates.^[^ ^107^ ^]^ The focus and development on the base of basic features like shapes and sizes of the metallic nanoscale materials have elevated the degree of applications in domain of nanophotonic. The tunability and adaptability of the localized resonance bands can be achieved by variations in these basic features and ultimately improving SERS efficiency.

To observe the effect of length and to obtain high SERS enhancement, Au/Ag nanorods have been synthesized with variating length ranging from 200 to 1200 nm (230, 450, 700, 800, 1000, 1200 nm) by utilizing Nano bi‐pyramidal Au core and Ag shell. The bi‐pyramidal Au core gave more help to tune LSPR as compared to spherical, or rod shape Au core.^[^ ^108^ ^]^ The relative SERS improvement proficiency of these core–shell gold@silver NRs has been estimated by utilizing 4‐mercaptobenzoic acid (4‐MBA), crystal violet (CV), and 4‐mercaptopyridine (4‐MPy) as Raman tag, and the obtained SERS results showed NRs with length of 450 nm enormous enhancement (∼10^10^ for 4‐MBA) among all compared NRs which was validated by limited difference time‐domain (FDTD) virtual experiences.^[^ ^109^ ^]^ Moreover, Ag NRs with similar lengths were prepared and compared with 450 nm core/shell Au/Ag NRs. The outcomes of this comparison exhibited that Au/Ag NRs had four times better SERS enhancement as compared to AgNRs with similar length due to their large surface area for adsorption of Raman reporters and especially high field electromagnetic field contribution. It was also observed that core/shell NRs showed far better mono‐dispersity as compared to Ag NRs.^[^ ^82^ ^]^

In a previously reported work, monodisperse Au@Ag bimetallic NRs were efficiently prepared with diverse silver shell thicknesses while considering and studying the influence of the silver shell thickness on their plasmonic features and SERS performance.^[^ ^110^ ^]^ In experiment, various monodisperse bimetallic Au@Ag nanorods were synthesized each with different thickness of silver shell. It was indicated that Au@Ag NRs had stronger SERS enhancement as compared to AuNRs and second, SERS activity enhanced more with increasing the Ag shell thickness. Besides, Silver NPs have been studied to contain shorter plasmonic wavelength comparison to Gold NPs due to their superior plasmonic features, but the thickness factor would upgrade and expand the applicability of silver NPs in optical applications. Due to this factor, blue‐shifted longitudinal plasmonic bands and transverse plasmonic bands of Au@AgNRs were achieved leading to explain the phenomenon of transverse‐dipolar‐plasmonic method.^[^ ^111^ ^]^

## 4 POTENTIAL APPLICATIONS

### 4.1 Viral detection

The emergence of several new biological threats around the world has caused a worldwide upsurge to identify new efficient and rapid methods for early identification and assessment of viral pathogens. Research community is making continuous efforts to develop potential, efficient, fast, and accurate procedures for the early diagnosis and assessment of dangerous pathogens in disease monitoring systems. Usually, PCR and ELISA based techniques are performed to detect viruses. However, these techniques have several major flaws: (i) time‐consuming, (ii) require a complex preliminary isolation procedure of the target species, (iii) require laborious incubation and purification stages, and (iv) limited to using highly specific antibodies or primers, making it impractical for on‐site rapid detection. Therefore, the recent application of surface‐enhanced Raman scattering (SERS) based biosensors was discovered to be ultra‐efficient, cheap, and rapid detection method to diagnose viral disease infection at early stages.^[^ ^112^ ^]^ Here in our review, we are focused on discussing the application of novel SERS‐active substrates based on Au–Ag nanostructures in the detection of viral diseases.

SERS based immunoassays employing different alloys and biomolecules were developed to detect Hepatitis B virus.^[^ ^112^, ^113^ ^]^ Recently, a highly sensitive SERS immunoassay using a Raman reported and the novel SERS based active substrate on microfluidic chip was developed to detect Hepatitis B virus.^[^ ^112e^ ^]^ Hepatitis disease was detected in human blood plasma by microfluidic chip carrying antibodies, known as Hepatitis B surface antigen (HBsAg). Raman reporter, basic fuchsin provided an effective SERS improvement and demonstrated excellent ability to joint with both Au nanostructures and antibodies. This fuchsin (FC) labeled gold nanoflowers immunoassay produced a sandwich like structure comprising of antigen and antibody. This sandwich structure was restrained on the SERS substrate made of Au–Ag coated gallium nitride (GaN) crystals via 6‐amino‐1‐hexanethiol (AHT) layer using EDC/NHS standard coupling chemistry. This SERS‐active substrate was proved to be critical in enhancing the efficiency of SERS immunoassays due to its high stability, reproducibility, and strong surface‐enhancement. This immunoassay was employed to diagnose the Hepatitis B virus in human blood plasma. For this purpose, calibration curve was established by associating the SERS signal strength of the spectral band at 1178 cm^−1^ with the concentration of antigen production. Furthermore, a limit of detection as 0.01IU/ml was estimated for HBsAg that proved high specificity for HBsAg detection.

The upper and lower respiratory tract infections (RTIs) including pneumonia, acute bronchitis, and acute tracheitis, etc. have been a major global health concern for decades. It has been very difficult to give the precise clinical diagnosis of RTIs as diverse pathogens could be the causative agents, that is, bacteria and atypical pathogens including influenza A and B, parainfluenza 1,2, and adenovirus. Moreover, late, or inaccurate diagnosis of RTIs can even lead to death of the patient. Hence, accurate, and early‐stage diagnosis of pathogen is fundamental for the treatment of patients. These RTIs often coexist and are already available technique for their identification such as immune‐fluorescence assay (IFA), based on the diagnosis of antibodies in human blood serum may give false positive results. Therefore, a lateral flow microarray based on SERS Nanotags was developed for rapid and ultrasensitive assessment of multiplex RTI pathogen.^[^ ^114^ ^]^ They fabricated a 2 × 3 microarray with SERS‐nanotags (Ag Methyl blue@Au and Ag Nile blue@Au) encoding the nucleic acids of 11 highly prevalent RTI pathogens. The limit of detection (LOD) for Influenza A, Influenza B, Parainfluenza 1, Parainfluenza 2, Parainfluenza 3, and Adenovirus was reported as 0.031, 0.035, 0.030, 0.032, and 0.040 pM, respectively.

SERS‐active substrate of Au/Ag multilayered was employed to develop a simple method to diagnose influenza A virus strain.^[^ ^112f^ ^,^ ^112g^ ^,^ ^115^ ^]^ Previously, SERS based immunosensors employed the flat substrate that is, gold or glass substrate.^[^ ^116^ ^]^ These flat substrates showed to cause low efficiency due to non‐significant enhancement of electric field. Hence, highly sensitive biosensor was developed by combining excellent signal enhancement properties of SERS with highly specific antigen/antibody interaction. These biosensors helped to detect the highly conserved, nucleoprotein of influenza A virus. SERS substrates were synthesized by gold nanoparticles labeled with 4, 4′‐thiobisbenzenethiol (TBBT) and PEG molecules and further immobilization with influenza A antibody. SERS probes immunoassay was conducted on 2D‐arrays of Au@Ag with outer‐surface of Au/Ag nanoparticles (NPs), which effectively improved the accuracy and sensitivity of detection. The selective nucleoprotein that is, antibody recognition was detected by using the TBBT SERS signal. These SERS substrates improved the detection power to ∼4 times as compared to flat gold (Au) substrates. This sensitivity and efficiency of SERS‐based biosensors were achieved by using effective fabrication of Au@Ag 2D array instead of a flat Au film. This technique provided the detection of influenza A virus with 6 TCID_50_/ml. Recently, another technique, SERS based antibody probes were employed for easy, rapid, and efficient detection of influenza A virus.^[^ ^112g^ ^]^ This technique used a simple way to synthesize the SERS based antibody without any complicated biological and chemical reactions. This probe fabrication was performed by simple mixing of gold nanoparticles, Au binding protein G, and antibody. These SERS based antibody probes provided the assessment limit of 4.1 × 10^3^ TCID/ml with efficient selective detection for influenza A virus (pH1N1).

During the last 2 years, COVID‐19 pandemic has been devastating human life worldwide. COVID‐19 (coronavirus disease 2019) is strain of SARS‐coronavirus 2 (severe acute respiratory syndrome‐Coronavirus 2). Novel COVID‐19 was found to be the threatful respiratory syndrome. COVID‐19 is a single stranded RNA virus, and its genomic length is about 30 kilobytes that also acts as a mRNA to perform immediate translation of viral proteins. There are fourteen open reading frames (ORFs) in the SARS‐coronavirus genome and the 2/3^rd^ of the 5´ genome is occupied by two ORFs; ORF1a and ORF1b, and encodes nonstructural proteins (NSPs). The 1/3^rd^ of 3´ genome synthesized the four structural proteins including spike, envelope, membrane, and nucleocapsid proteins. The spike protein has normally two subunits, S1 and S2, and has a key role in binding virus to receptors prevailed on the host cell surface. Therefore, receptor binding domain (RBD) is essential for binding SARS‐coronavirus, therefore, it becomes one of the potential therapeutic targets in detecting the COVID‐19. In this regard, SERS based novel approaches were implemented for disease monitoring, early assessment of disease, disease monitoring, and the production of vaccines.^[^ ^114^, ^117^ ^]^

COVID‐19 surveillance at the population level necessitates swift and reliable tests to reduce community transmissions. The detection of volatile organic molecules in the breath is a potential alternative, but it is currently hampered by large gear and a rigid analysis process. SERS‐based breathalyzer takes advantage of major changes in vibrational signatures caused by interactions between breath compounds and various molecular receptors to develop a strong partial least squares discriminant analysis model for high throughput classifications.^[^ ^118^ ^]^ Northern Arizona University Professor Miguel Jose Yacaman and his research team created a novel non‐invasive diagnostic process that used nanotechnology, plasmonic, and two‐dimensional materials for the quick detection of the spike proteins of the SARS‐CoV‐2 virus using the single‐molecule surface‐enhanced Raman spectroscopy technique.^[^ ^119^ ^]^ Israeli researchers also created a 1‐min breath analyzer based on spectroscopy to identify coronavirus. They used hybrid nanomaterial sensors to make the breath analyzer for the detection of SARS‐CoV‐2 from volatile organic molecules present in the exhaled air. The breath analyzer detected Covid biomarkers in the expired breath with the accuracy of up to 92 percent, the sensitivity of up to 100 percent, and specificity of up to 84 percent in controlled clinical studies conducted in March 2020 at Wuhan.^[^ ^120^ ^]^

Similarly, Carlomango and his colleagues used saliva samples from patients with coronavirus disease to develop a Raman spectroscopy‐based detection methodology. Their Raman fingerprint data revealed significant proof of infection by providing a biochemical signature of COVID‐19‐infected saliva samples and developed a novel, quick, and non‐invasive detection technique that proved to be highly accurate, precise, specific, and sensitive.^[^ ^121^ ^]^ Sanchez et al. also used the SERS technique to detect SARS‐CoV‐2 and the specific coronavirus S and N proteins.^[^ ^122^ ^]^ Recently, Zhang et al. created an assay that used SERS in conjunction with multivariate analysis for the identification of the SARS‐CoV‐2 in real‐time, with no additional preprocessing. They used Ag nanorod as a SERS array that was aided with the ACE2 receptors. The robust SERS signals of the ACE2 were measured at the wavelengths of 1032, 1051, 1089, 1189, 1447, and 1527 cm^−1^.^[^ ^123^ ^]^ Recently, another novel label‐free SERS biosensing technique has been published that used metallic nanostructures to determine the efficiency of the vaccine Oxford‐AstraZeneca (AZD1222) in the patients that were earlier vaccinated. As a ChAdOx1 vector of chimp adenovirus that is unable to replicate, but used to generate the AstraZeneca vaccine, it validated the prediction that the tears of a person who is vaccinated with AstraZeneca may be comparable to those of keratoconjunctivitis patients that is another disease caused by adenovirus. Furthermore, it demonstrated the potential of up to three biomarkers for predicting the status of vaccination by the measurement of the signals emitted by the antibodies using this approach with great consistency.^[^ ^124^ ^]^

**FIGURE 1:**
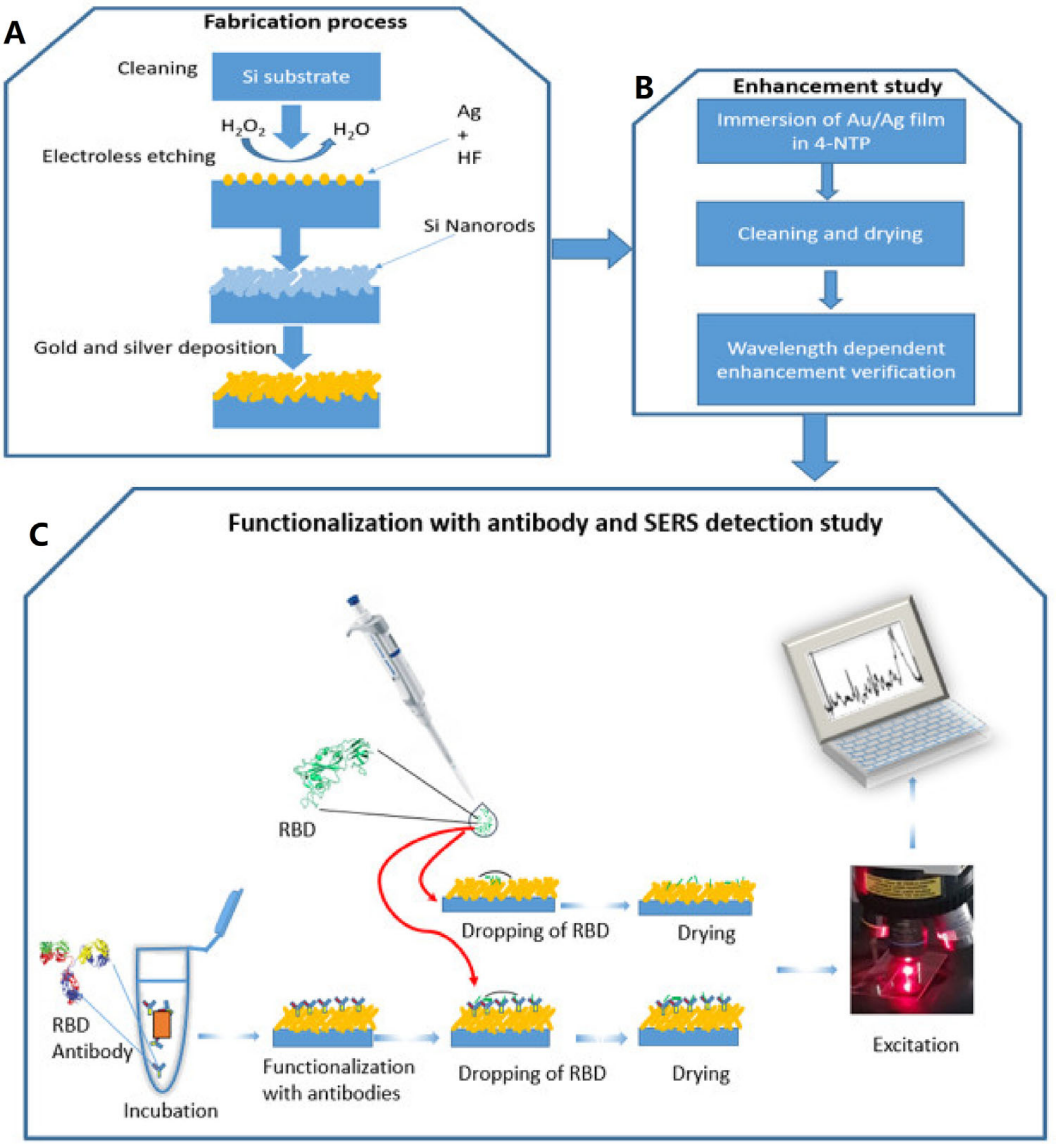
A schematic representation of three processes: (A) fabrication process, (B) enhancement study, (C) functionalization with antibody and receptor binding domain detection by SERS. Reproduced under the terms of the CC BY 4.0 license.^[^ ^117c^ ^]^ Copyright 2021, The Authors.

Awada and his colleagues developed a unique method for detecting the RBD protein of COVID using the SERS technique (Figure 1). They fabricated Au/Ag nanostructures substrate that showed high efficiency and accuracy to observe the RBD protein in a short time about 3 s. This approach reported a limit of detection (LOD) of proteins to single protein detection of 1 pM.^[^ ^117c^ ^]^ Another novel approach was developed that enabled the rapid and sensitive detection of SARS corona virus antibodies.^[^ ^117d^ ^]^ In this method, they fabricated the MoS2 field effect transistor for efficient assessment of antibodies in SARS‐CoV2 receptor binding protein from vaccinated blood serum. A pioneer SERS based biosensor was fabricated for COVID ultra‐efficient detection from saliva without prior treatment.^[^ ^114^ ^]^

### 4.2 Bacterial detection

Human health and safety have always been at risk due to pathogenic bacteria. They may cause some serious and life‐threatening infections even at a low infectious dose.^[^ ^125^ ^]^ Many different strategies have been developed for the detection of bacteria.^[^ ^126^ ^]^ Currently, two different SERS techniques have been in use for bacterial detection. First is a label‐based SERS detection technique that uses SERS‐tags. Second one is a label free SERS detection technique. This approach does not need any target label, and can directly detect bacterial pathogens from their intrinsic vibrational fingerprints. This label‐free SERS detection technique is easier, rapider, and more efficient as compared to the label‐based SERS technique. A tag free SERS detection approach, named as, “capture enrichment enhancement (CEE)” was reported for achieving rapid bacterial assessment (within 10 min) in tap water and milk samples.^[^ ^127^ ^]^ This SERS was fabricated by combining the polyethyleneimine, gold‐coated magnetic microspheres (Fe_3_O_4_@Au), and gold@silver nanoparticles. The positive charge of Fe_3_O_4_@Au@PEI SERS microspheres quickly captured the negative charge bacteria in the desired solution. Gram‐positive bacterium *Escherichia coli* and *Staphylococcus aureus* were detected by this CEE method and limit of the detection at 10^3^ cells/ml was reported. This label‐free SERS platform has a lot of potential in the field for environmental monitoring, food safety, and biological threat detection.

Another new and natural technique was developed for the efficient assessment of the bacteria. In this approach, large scale three‐dimensional super‐crystal was fabricated by using Au@Ag NPs labeled on mussel shell.^[^ ^128^ ^]^ It was learned that the higher concentration of calcium carbonate in mussel shell and nanoparticle channels together could make the SERS efficient and rapid detection. Nanoparticles on the surface compared to nanoparticles coated in the channel provided the higher electromagnetic improvement. This strategy improved the efficiency and detection limit of SERS substrate 10^–9^ M. This method could potentially be utilized in the sensitive detection and discrimination of *S. aureus*, *E. coli* and *Pseudomonas aeruginosa* Au@Ag nanorods with two differ distinctive SPR at 400 and 800 nm were synthesized and reported which induce linear fluorescence signals to detect *E. coli* and *S. aureus*.^[^ ^129^ ^]^

**FIGURE 2:**
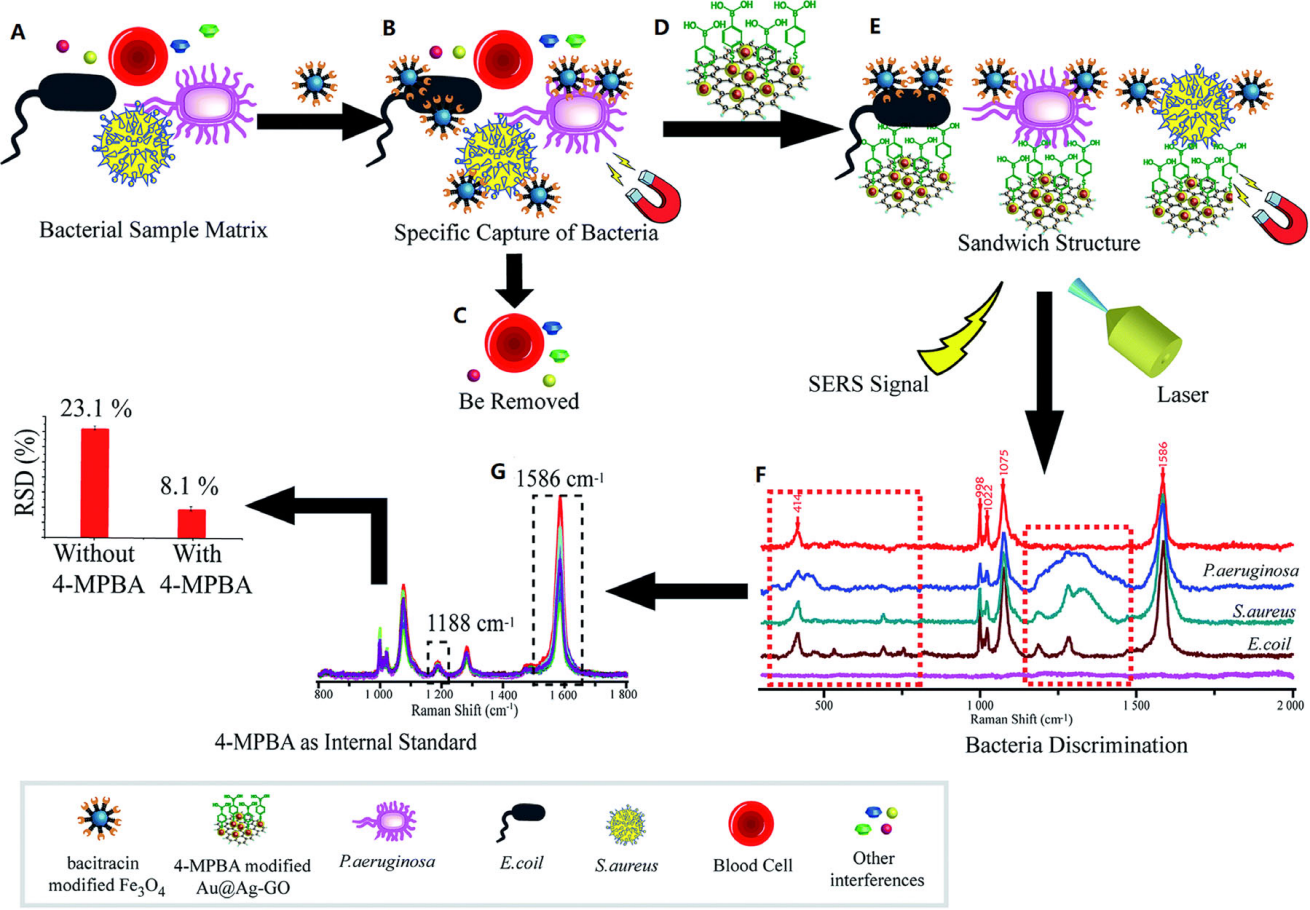
Schematic illustration of the operating procedures for bacterial detection via a SERS sandwich strategy, in which AMP modified magnetic Fe_3_O_4_ NPs were utilized in the bacteria capture and 4‐MPBA modified Au@Ag‐GO nanocomposites were used as SERS tags. (A) AMP modified Fe_3_O_4_ NPs were cultured with a bacterial sample matrix, which included bacteria, blood cells, or other interference. (B) The Fe_3_O_4_ NPs@bacteria complex was magnetically separated from the sample matrix. (C) Blood cells or any other interference were removed. (D) 4‐MPBA modified Au@Ag‐GO nanocomposite SERS tags were cultured with the Fe_3_O_4_ NPs@bacteria complex to form a sandwich structure. (E) The Fe_3_O_4_ NPs/bacteria/SERS tags sandwich structure was magnetically separated and detected by the Raman spectrometer. (F) Different kinds of bacteria were discriminated according to their Raman “fingerprints.” (G) 4‐MPBA can be used as an IS to correct the SERS intensities. Reproduced under the terms of the CC BY‐NC 3.0 license.^[^ ^130^ ^]^ Copyright 2018, Kaisong Yuan et al.

A novel biosensor was developed based on sandwich structure that could be used to isolate and detect multiple pathogens of bacteria through SERS probes and magnetic separation.^[^ ^130^ ^]^ This bioassay relied on anti‐microbial peptide functionalized magnetic nanopores for detecting and isolating bacterial pathogens and Au coated Ag labeled graphene oxide (GO) nanocomposites (Au@Ag‐GO). Further, SERS probes were modified with four‐mercaptophenylboronic acid (4‐MPBA) (Figure 2). These SERS‐tags were combined with various bacterial pathogens. 4‐MPBA fingerprints showed corresponding changes due to their interaction with different kinds of bacterial cell walls and successfully isolated and discriminated three different bacterial pathogens including *E. coli*, *P. aeruginosa*, and *S. aureus*, with lowest concentration of 10^1^ CFU/ml for each of the three strains. Furthermore, the AMP modified Fe_3_O_4_ NPs showed strong antibacterial activity with low cellular toxicity and therefore, could act as antibacterial agents in long‐term blood preservation for safe blood transfusion applications. In a nutshell, this multifunctional biosensor had the potential to isolate, discriminate and destroy bacteria all at the same time, indicating its great prospective for clinical diagnostics and protected blood transfusions.

Recently in a research work, a SERS substrate Au@Ag core–shell nanorods (NRs) tags were applied for food based bacterial pathogen assessment and antibiotic susceptibility analysis.^[^ ^131^ ^]^ They fabricated Au@AgNR@SA by functionalizing Au@AgNRs with Raman label and streptavidin. Later, this complex was linked with biotinylated antibodies through strong noncovalent interactions. These SERS substrate nanotags (Au@AgNR@SA@Ab), could particularly capture and enrich *E. coli* from sample. These SERS‐active nanotags (Au@AgNR@SA@Ab) identified *E. coli* with a lowest limit of detection as 10^2^ CFU/ml. In the next step, *E. coli* were exposed to ampicillin at an MIC of 4 µg/ml. Raman spectroscopy was applied to discriminate accurately and swiftly between ampicillin susceptible *E. coli* and ampicillin resistant *E. coli* (Ampþ‐*E. coli*) within 3.5 h.

Recently, a novel SERS‐based immunocapture nanoprobe was developed for pathogenic bacterial detection.^[^ ^132^ ^]^ Polymerized dopamine (PDA) was coated on Au@Ag NPs for stabilizing SERS signals and protecting these noble metals from chemical oxidation. Then, these Au@Ag@PDA nanoparticles were functionalized with boronic acid (RB(OH)_2_) that aided in their binding property to pathogenic bacteria and induced signal amplification. In this technique, IgG@Fe_3_O_4_ nanoparticles served as a magnetic separation agent, and the SERS tag was employed to enhance the Raman scattering. The Raman signals were enhanced to 10^8^ times after these SERS tags were bound to the bacterial surface. Bacterial classification of *E. coli*, *Klebsiella pneumonia*, *P. aeruginosa*, *S. aureus*, and *Shigella dysenteriea* became possible due to specific fingerprint‐like patterns of spectral lines. LOD for this approach was low as 10 CFU/ml. This method was limited to bacteria with high binding affinity for IgG.

**FIGURE 3:**
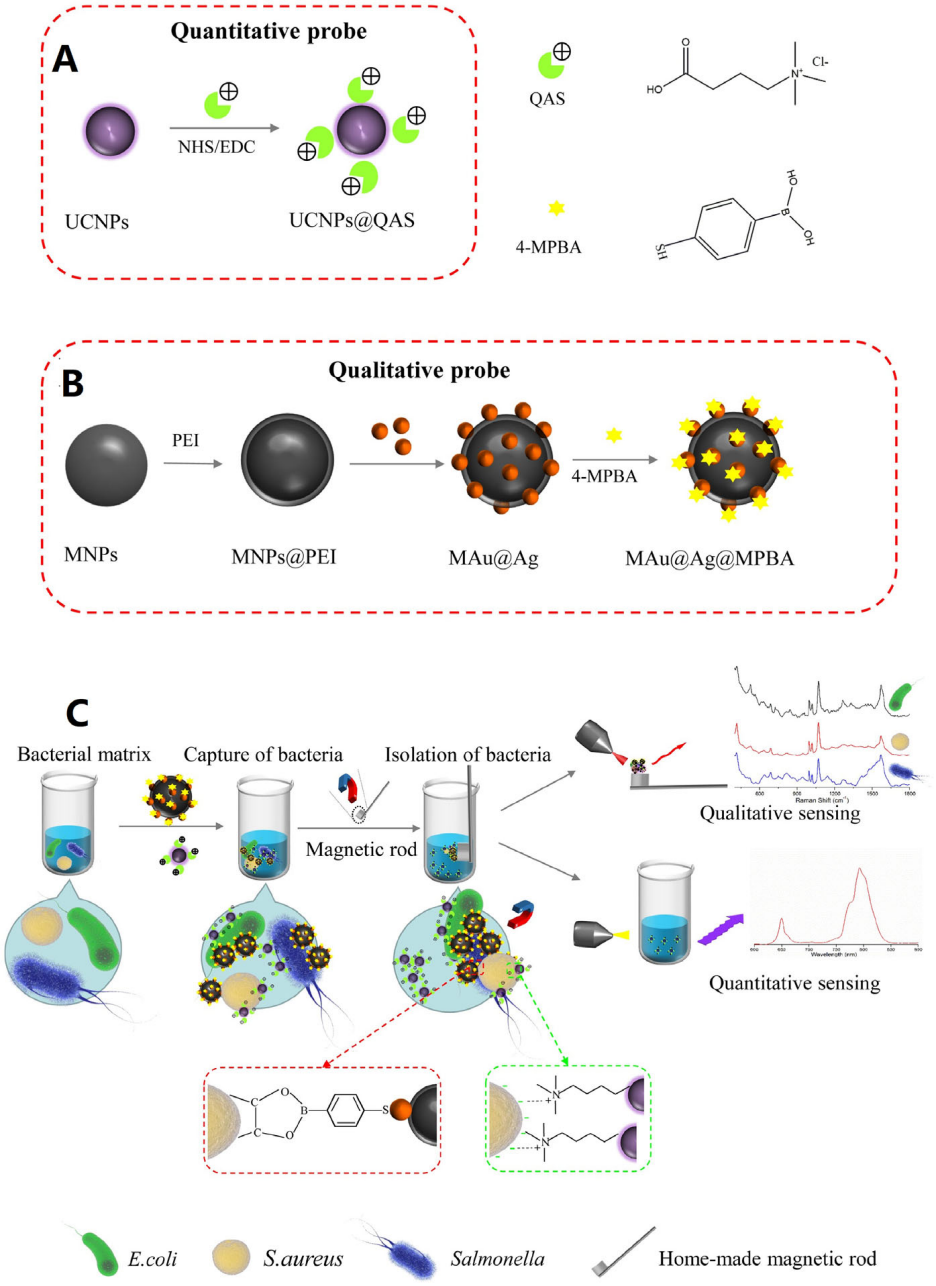
Schematic description of the proposed integrated multiple nano‐platform. (A) Fabrication of UCNPs@QAS by grafting quaternary ammonium salt (QAS) onto up‐conversion nanoparticles (UCNPs) via an amidation reaction. (B) Fabrication process of MAu@Ag@MPBA. (C) Isolation and detection of *E. coli*, *S. aureus*, and *Salmonella* via dual‐mode sensing. Reproduced with permission.^[^ ^134^ ^]^ Copyright 2021, Elsevier

In recent years, optical biosensor techniques that rely on the assessment of scattering, absorbance, and fluorescence have been used as ideal analytical tool for bacterial discrimination, owing to their sensitivity, ease of operation, and low cost. Particularly in last few years, SERS substrate and up‐conversion nanoparticles (UCNPs) based fluorescence approach has intrigued the attention of research community.^[^ ^133^ ^]^ In a recent study, multifunctional biosensor platform was reported that was based on combining SERS and UNCPs‐based fluorescence technology to investigate total number of bacteria and discriminate between types of bacteria simultaneously. In this detection system, Au@Ag nanoparticles were developed with four‐MPBA for regulating the SERS probe signals of pathogenic bacteria for accurate assessment and detection. Quaternary ammonium salt (QAS) modified UCNPs were also synthesized to capture the bacteria present in food sample and to identify the total bacterial count.^[^ ^134^ ^]^ The dual mode Au@AgNRs and UCNPs@QAS was used for classification, isolation, and observed the total number of bacteria (Figure 3).

Another important bacterial biomarker discovered is pyocyanin, that is in fact a toxin released by a problematic infectious gram‐negative bacterium *P. aeruginosa*.^[^ ^135^ ^]^ This bacterium has an important role in its gene‐expression. Pyocyanin is supposed to be a virulence element and least sensing signal for *P. aeruginosa*. This is an outstanding biomarker for early diagnosis of RTIs. In a recent study, Au@Ag nano‐star (NSs) dimers were fabricated on DNA origami. Bimetallic nano‐stars of 70 nm Au@Ag NSs were synthesized on rectangular DNA origami with a mean interparticle space size (10 nm). These SERS probes were reported for the ultra‐sensitive detection and rapid label free diagnosis of pyocyanin. SERS probe spectrum revealed distinct bands of pyocyanin even at very low concentration of 335 pM. Thus, it is potentially promising rapid, label free, and extremely sensitive method for the assessment of pyocyanin leading to early‐stage diagnosis of severe *P. aeruginosa* infections.

Bacterial contamination is common in many fields, including the aquaculture business. Pathogenic bacteria of many types can cause virulent epidemic diseases, which is ascribed to the 1/3^rd^ of global fish mortality each year.^[^ ^136^ ^]^ High‐density aquacultural approach can cause the outbreak of virulent diseases due to hazardous fish bacteria. Therefore, pathogen‐prevention assessment is required to be implemented for early and accurate diagnosis of the disease. In this regard, better fish disease management, more unique, reliable, and fast methods of evaluating the fish bacteria have to be devised. For this purpose, unique and label free SERS substrate approach was employed for the early and fast detection of fish bacteria including *Edwardsiella piscicida*, *E. coli*, *Vibrio anguillarum*, *Vibrio harveyi*, and *Pseudomonas plecoglossicida* ^[^ 136 ^]^. In this study, Fe_3_O_4_ magnetic nanospheres were combined with polyethylenimine (PEI) to modify the surface charge of Fe_3_O_4_ magnetic nanospheres making them effective in capturing negatively charged bacteria within 10 min. Then, Au@Ag core–shell bimetallic nanoparticles provided major hotspots that resulted in strong Raman signal enhancement. This approach provided the limit of detection (LOD) as low as 10^5^ CFU/ml.

**FIGURE 4:**
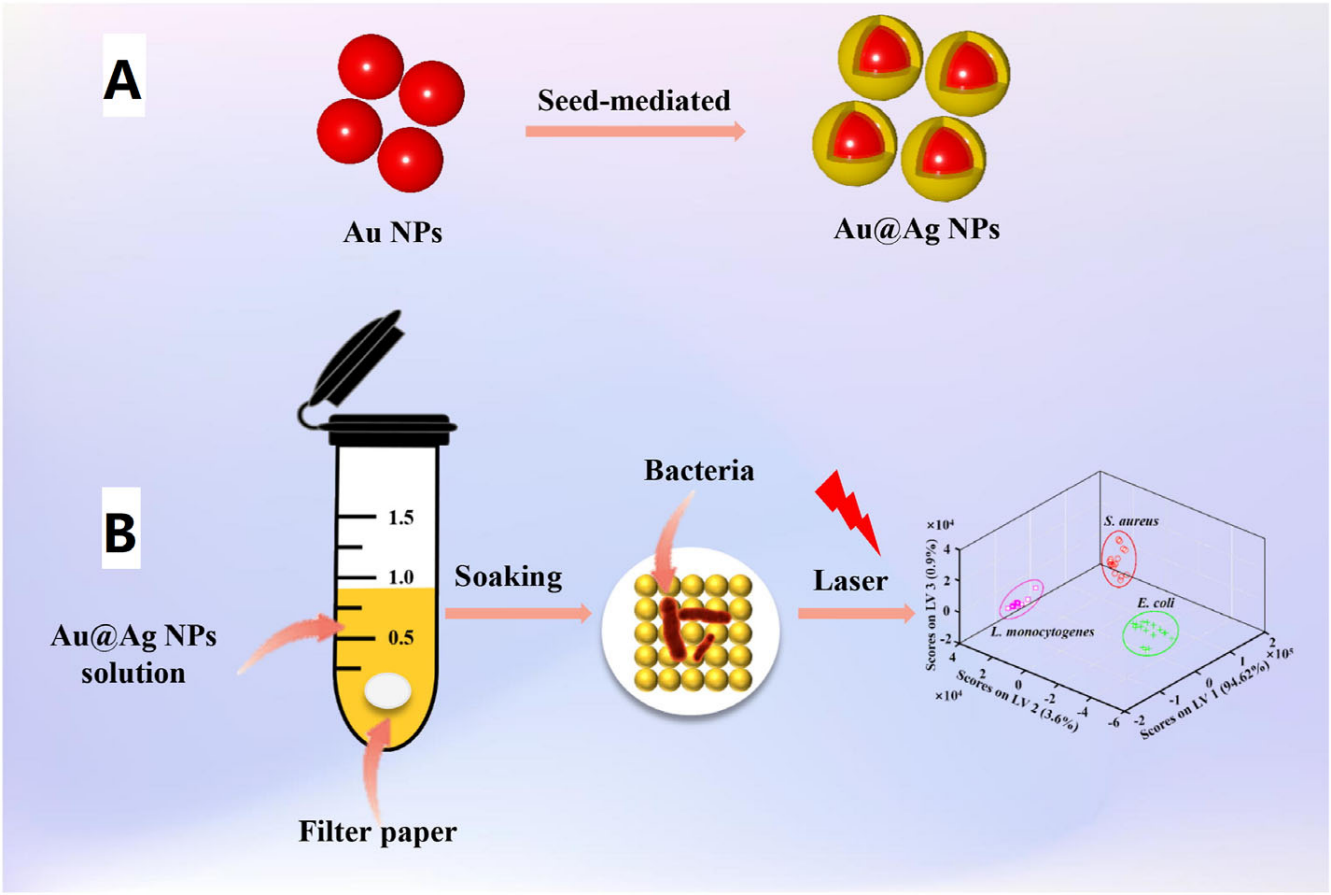
Schematic illustration of the proposed SERS sensor based on Au@Ag NPs solid‐phase substrate. (A) Preparation of Au@Ag NPs by seed‐mediated two‐step growth method. (B) Bacterial SERS spectral processing and quantification. Reproduced with permission.^[^ ^137^ ^]^ Copyright 2022, Elsevier

In one other new work, seed growth method was used to fabricate Au@Ag NPs that were self‐synthesized on filter paper sheets employing dip coating technique.^[^ ^137^ ^]^ This SERS substrate is known as filter paper‐based SERS probes (Figure 4). The excellent absorbance of filter paper restrained the bacteria on SERS probes, and enhanced the connection and detection signals between the bacteria and SERS probe. Suspensions containing three food borne bacteria, including *E. coli*, *S. aureus*, and *Listeria monocytogenes*, were poured dropwise to the filter paper‐based SERS substrate for SERS signal assessment. The effective identification of these food borne pathogens was done by combining partial least squares‐discriminant analysis (PLS‐DA) with bacterial SERS spectra observations.

### 4.3 Circulating tumor cells and living cancer cells detection

Primary tumor releases rare tumor cells into the blood circulation known as circulating tumor cells or CTCs.^[^ ^138^ ^]^ These CTCs are enriched with molecular information like gene and protein expression and this data is fundamental in providing insight into the metastasis and invasiveness of cancer.^[^ ^139^ ^]^ It is evident from clinical studies that the number of CTCs in peripheral blood of a cancer patient can tell a lot about the survival rate and treatment monitoring of the cancer patient and therefore, CTCs are used as liquid biomarkers for assessing the metastasis, relapse, and prognosis of cancer.^[^ ^140^ ^]^

For early diagnosis and effective treatment of liver cancer, Pang et al., developed magnetically assisted SERS biosensor to detect hepatocellular carcinoma (HCC) CTCs from blood samples. The biosensor was comprised of two different elements including anti‐ASGPR antibody‐Fe_3_O_4_@Ag MNPs and anti‐GPC3 antibody‐Au@Ag@DTNB NRs. This system showed good performance in real system and a LOD of 1 cell/ml for HCC CTCs in human peripheral blood samples was achieved as both MNPs Ag shell and the Au@Ag NRs SERS tags provided dual enhancement and both anti‐ASGPR and anti‐GPC3 antibodies provided dual selectivity.^[^ ^141^ ^]^

**FIGURE 5:**
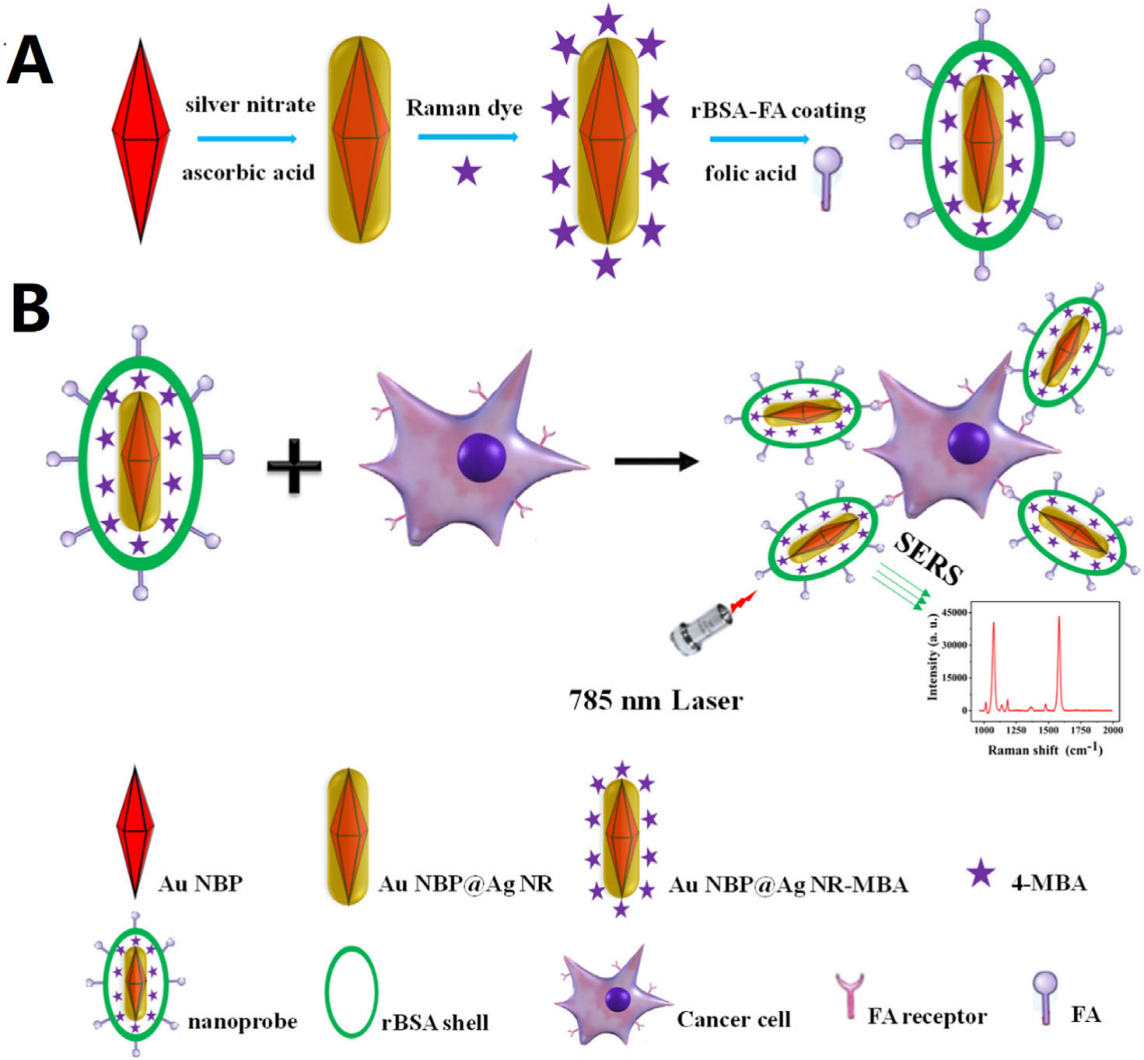
Illustration of Au NBP@Ag NR‐MBA‐rBSA‐FA nanoprobes for SERS detection of living cancer cell. (A) Fabrication of Au NBP @ Ag NR‐MBA‐rBSA‐FA nanoprobes; (B) target cancer cells detection. Reproduced with permission.^[^ ^142^ ^]^ Copyright 2019, Elsevier

Living cancerous cells identification is tremendously critical for disease prediction, early diagnosis, and therapeutic efficacy of cancer. However, development of an effective method for fast identification and bioimaging of living cancerous cells is an urgent matter. Recently, Chang et al. reported a simple and time‐saving method for producing Au NBP@Ag nanorods core–shell NPs with excellent mono‐dispersity by growing the silver shell directly on gold NBP core surface. These core–shell NPs exhibited high SERS activity due to good responsibility, favorable uniformity, and high stability. For rapid detection of living cancerous cells in cell plates, Chang et al. fabricated SERS‐active Au NBP@Ag, NR‐MBA‐rBSA‐FA nanoprobes that possessed excellent SERS activity, excellent detection efficiency, and good stability under optimal conditions. These nanoprobes efficiently detected MGC‐803 cells with excellent reproducibility through SERS‐mapping and obtained Raman mapping images with high resolution. The high sensitivity and excellent specificity of these developed nanoprobes could be confirmed by comparing the Raman mapping images of MGC‐803 with A549^[^ ^142^ ^]^ (Figure 5).

Core–shell plasmonic nanorods (PNRs) are preferable because they provide a uniform and strong magnetic field, tunable morphologies, LSPR resonance, and a stable SERS intensity. PNRs core–shell with tunable nanogaps was fabricated through coordinated interactions and galvanic replacement reaction. Later, 4‐mercaptopyridine (4‐MP) was coated on Au NR core surface, and a pyridine‐Ag^+^ coordination complex was formed, which aided in peripheral Ag shell growth. The synthesized PNRs amplified the intensity of Raman signals, producing an excellent quantitative SERS technique with high stability. Then 4‐mp was replaced with rhodamine and again PNRs with uniform nanogaps were fabricated, and it further validated this synthetic mechanism. SERS quantification analysis of MCF‐7 cells was successfully done by using these PNRs and a fast, ultralow detection of 20 CTCs per thousand WBCs was reported in a blood mimicking fluid. SERS signals were not reported when the HEK and HELA cancer cells were added to the same fluid, which further verified the selectivity of this SERS platform.^[^ ^143^ ^]^

Circulating tumor cells (CTCs) can be used as a marker for finding the probability of cancer recurrence, patient's survival time, and treatment efficacy. Lately, a highly specific multiplex targeting approach was developed for the kaleidoscopic identification of CTCs in human blood, using Au Nanorods coated with Ag (AuNRs/Ag). The spectral identification of CTCs was enhanced by excellent absorption and plasmonic Raman signals amplification by AuNRs/Ag on narrow SERS spectral‐lines and spectral PT signatures. These Au/Ag NRs were functionalized with four different Raman reporters and four antibody cancer biomarkers including anti‐IGF‐1 Receptor β, anti‐EpCAM, anti‐CD44, anti‐Keratin, and another specific WBCs biomarker anti‐CD45. Furthermore, these multimodal NPs simultaneously showed multicolored SERS and PT‐based high image resolution.^[^ ^144^ ^]^

**FIGURE 6:**
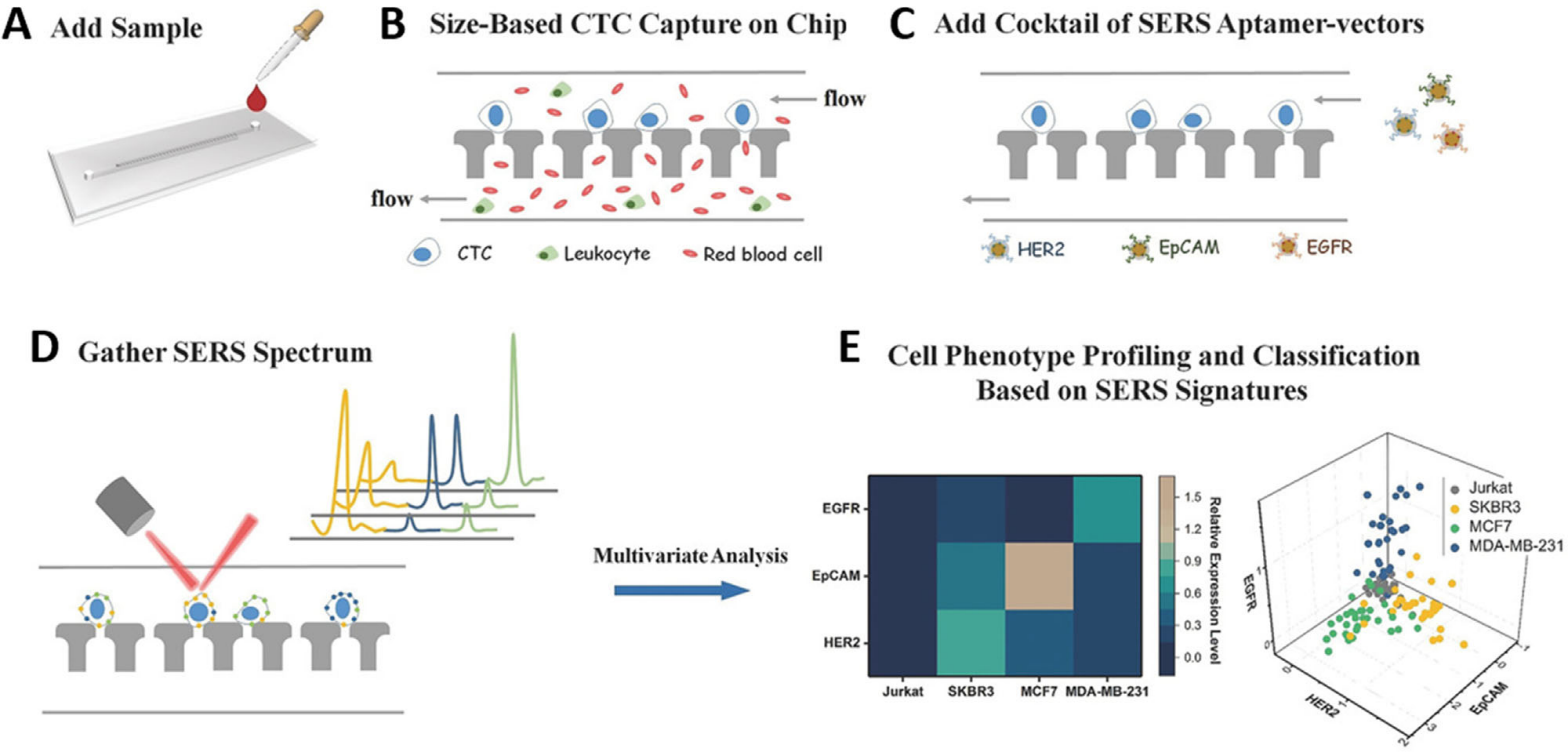
The workflow of operating on the platform for (A) add samples, (B) circulating tumor cells capture, (C) add SERS aptamer‐vectors, (D) gather SRES spectrum, (E) profiling of cell phenotype classification based on SERS signatures. Reproduced with permission.^[^ ^145^ ^]^ Copyright 2018, John Wiley & Sons

CTCs have heterogenous phenotype and in an individual, there may be a different subpopulation of CTCs existing that could be responsible for accelerating metastasis. In another study, a novel a microchip has been fabricated using the SERS‐based approach for in situ isolation, expression level profiling, and identification of multiple surface biomarkers of CTCs breast cancer subtypes. As an example, three different breast cancer cells were selected, and a microfluidic filter isolated the CTCs from human blood, based on the size differences between various blood cells and CTCs. Then the phenotypic data of captured CTCs was gathered easily by using spectrally orthogonal SERS probes that were based on their combined spectral signatures by utilizing least square algorithm strategy. The highly sensitive, selective, and reliable classification of several subtypes of human breast cancer cells could be done by combining this technique with the PLS‐DA classification algorithm^[^ ^145^ ^]^ (Figure 6).

SERS nanoprobes consisting Au@Ag core@shell NPs labeled with poly(allylamine) (PAH) molecules were fabricated for the simultaneous evaluation of expression level of two cancer‐related markers in cells and tissues. These Au@Ag NPs were combined with resonant Raman‐active substances, and as a result, a strong enhancement of SERS signal in the visible range was recorded. The PAH coating enabled specific biofunctionalization via its amine group and protected the metallic surface from chemical reduction, providing high stability in Raman signals. Two different nanoprobes were fabricated using tiny target molecules coated with polymer. Each nanoprobe was specific to a single cancer biomarker, one targeted the folate receptors (FRs), while the other targeted the sialic acid (SA). The expression levels of these targeted biomarkers (FRs and SA) were evaluated by measuring the SERS signal of probe in tissue samples of breast and ovarian cancerous. The overexpression of FRs and SA in tumor region was visualized with high contrast as compared to normal expression in other regions, and the structural localization of the tumor consistent with histology was achieved with high accuracy by SERS imaging.^[^ ^146^ ^]^

#### 4.3.1 Detection of multiple cancer cell biomarkers

Breast cancer is one of the major types of cancers reported worldwide and has been a major cause of death in females, therefore, early diagnosis of breast cancer is the critical need of time. For this purpose, ultrasensitive SERS‐biosensors were reported that were able to detect multiple biomarkers present on breast cancer cell. In this approach, PEGylated Ag‐encapsulated Au (Ag–Au) hollow nanospheres were proposed for the surface electromagnetic enhancement of Raman reporter molecules. For the detection of multiple protein biomarkers expressed on the surface of breast cancer cells, high stable and reproduceable SERS nanotags were designed. Raman reporter molecules (RBITC, MGITC, or DTDC) were positioned in nanogaps between the hollow Au nanospheres and Ag shell to achieve strong signal enhancements. Accurate identification of three breast cancer cell biomarkers including EpCAM, ErbB2, and CD44 was achieved by using PEGylated Ag–Au hollow nanospheres based on Raman imaging.^[^ ^147^ ^]^

**FIGURE 7:**
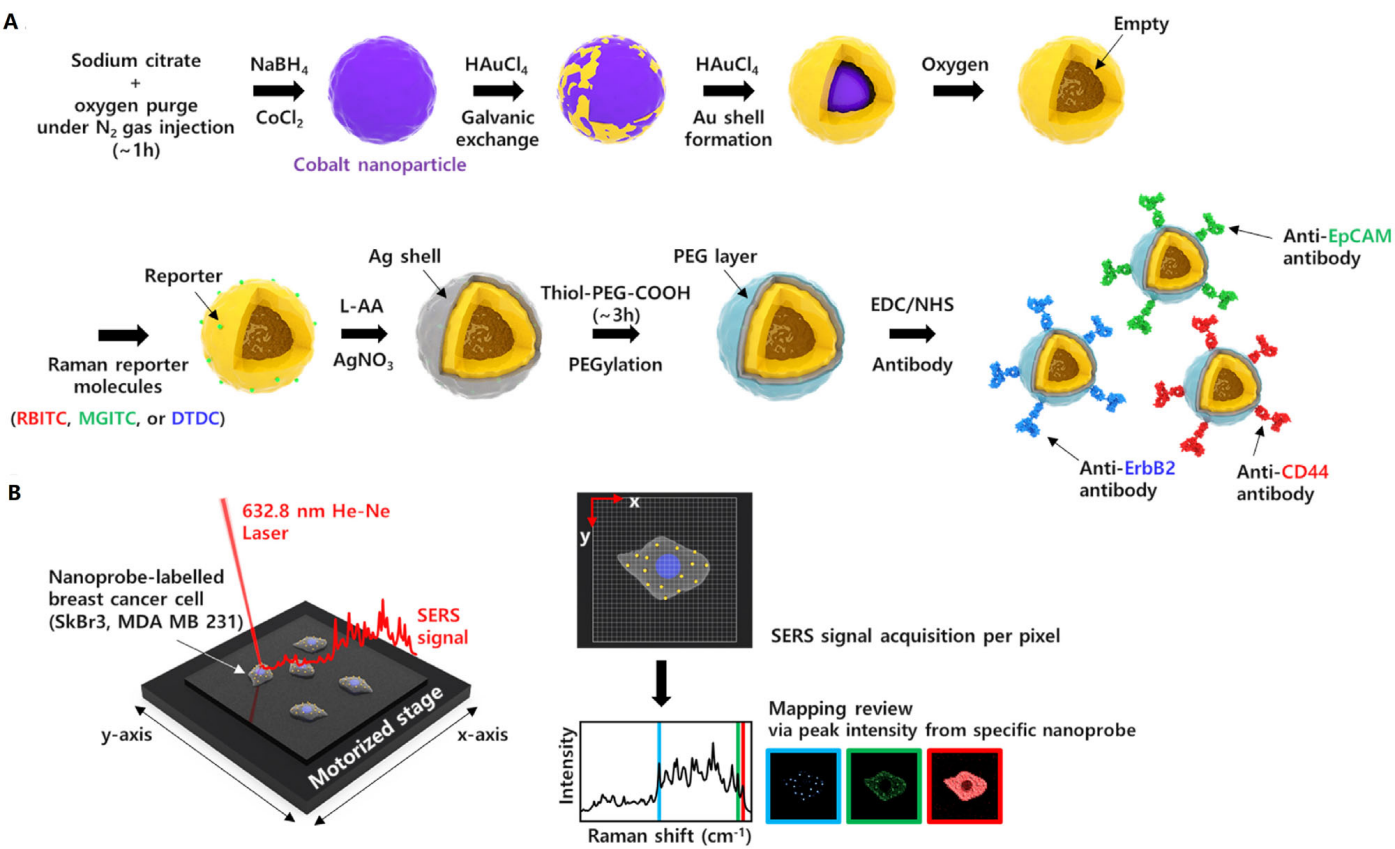
Schematic illustrations (A) for the fabrication of three different PEGylated Ag–Au hollow nanospheres which are environmentally stable under various pH values, temperatures, and salt concentrations, and (B) for simultaneous detection of three different biomarkers expressed in breast cancer cells using SERS mapping techniques. Reproduced with permission.^[^ ^147^ ^]^ Copyright 2020, Elsevier

Another approach was presented to detect multiple breast cancer cell biomarkers including CD63, HER2, and EpCAM using Hydrophobic Plasmonic Nanoacorn Array (HANA) aptasensor that was synthesized by etching hydrophilic patches array on the surface of a hydrophobic plasmonic substrate to improve the reproducibility and reduce the nonspecific binding. As a result, HER2 concentration from 1 fg/ml to 100 ng/ml was quantified in samples with RSD down to 5.2% using this rapid and ultrasensitive HANA technique^[^ ^148^ ^]^ (Figure 7).

#### 4.3.2 Detection of exosome like vesicles (ELVs)

ELVs are specific nanovectors released by a cell that contain various molecules like proteins, nucleic acids, or any other chemicals indicating molecular signature of a certain type of cell. A colloidal strategy for the identification of ELVs was reported that used Au@AgNPs core–shell system for reliable detection of individual ELVs, which suggested the differences in molecular composition of different types of cells. Furthermore, the acquisition time for this detection was decreased by a factor of 20 because of the strong enhancements generated by the core–shell system of the biosensor.^[^ ^149^ ^]^

### 4.4 SERS‐lateral flow assay

Lateral flow assay (LFA) is quite simple, easy, robust, and efficient analytical approach for point‐of‐care‐testing (POCT) and the detection of multiple types of proteins, viruses, pesticides, and other chemicals. However, a few drawbacks including low signal intensity as well as semi quantitative results have been reported in case of LFA, and various techniques have been combined with LFA to improve its sensitivity and quantitative analysis. One of those dual‐platform techniques is SERS‐based LFA that can significantly enhance detection and is much more convenient, sensitive, and reliable.^[^ ^150^ ^]^ For Example, a SERS‐based LFA biosensor was reported to analyze target DNA linked with Kaposi's sarcoma‐related herpesvirus of 0.043 pM and bacillary angiomatosis of 0.074 pM as their LOD.^[^ ^151^ ^]^ Similarly, a related SERS‐based LFA biosensor was proposed for the detection of C‐reactive protein with a LOD of 0.01 ng/ml.^[^ ^152^ ^]^

A similar SERS‐LFA platform was designed with easy, sensitive, rapid, and accurate detection of prostate cancer biomarkers including PDGF‐BB and thrombin. In this approach, SERS tags were created with Au/Ag HNPs that were modified with two different Raman molecules (DTNB and Nile blue A), and linked with a mouse monoclonal antibody and detection aptamers of PDGF and thrombin. This POCT approach allowed fast, ultrasensitive, and highly accurate detection of prostate cancer biomarkers PDGF‐BB and thrombin with very low LOD of 4.837 and 3.802 pg/ml, respectively.^[^ ^153^ ^]^

**FIGURE 8:**
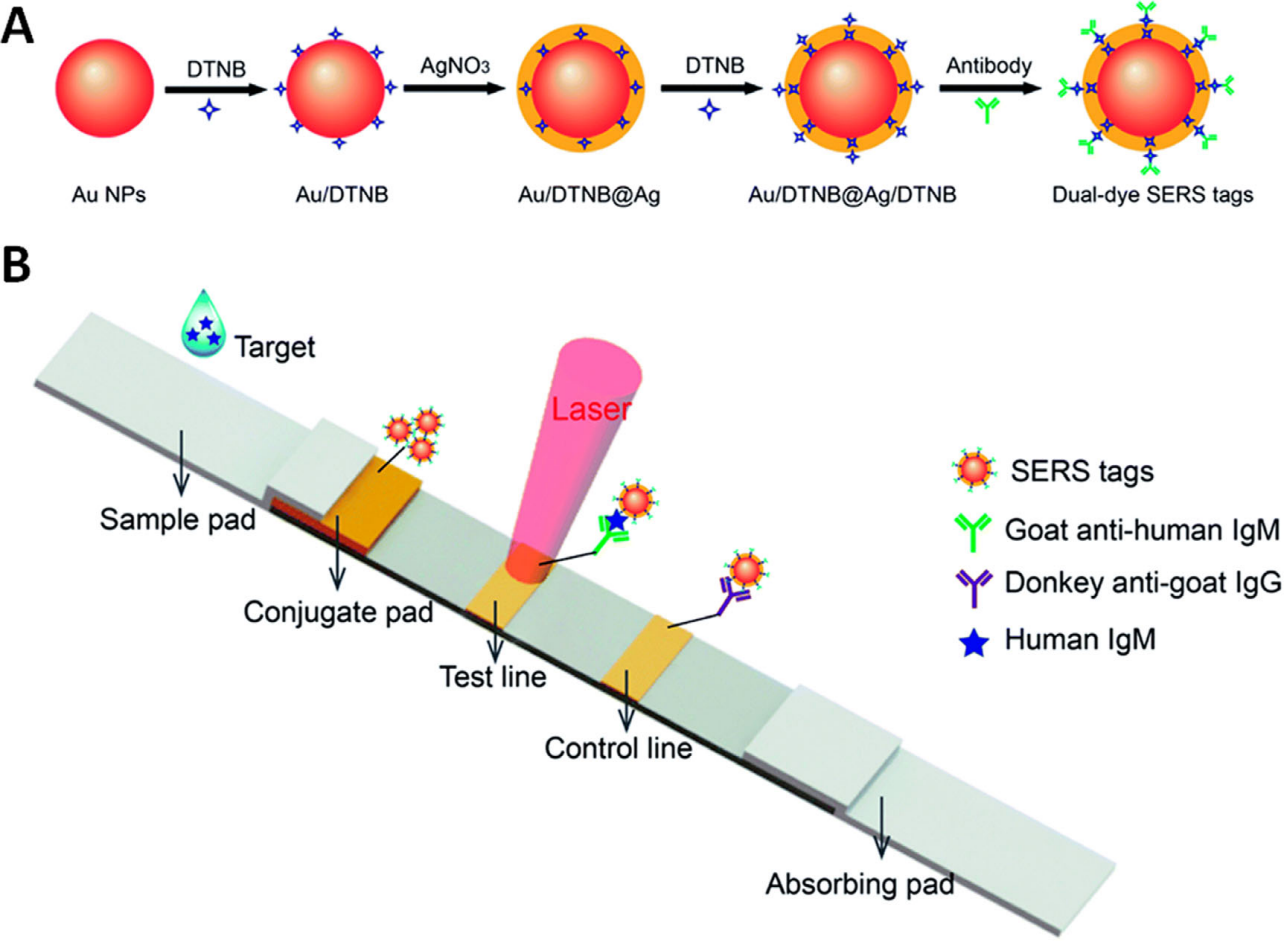
(A) Synthetic route for dual dye‐loaded SERS tags. (B) Schematic illustration of quantitative detection of human IgM using SERS‐based. Reproduced under the terms of the CC BY 3.0 license.^[^ ^154^ ^]^ Copyright 2018, Xiaofei Jia et al.

Another, SERS‐based LFIA has been presented to detect Mycoplasma pneumoniae (MP) infection in blood with high sensitivity and accuracy. This SERS‐LFIA strip consisted of Au@Ag NPs loaded with a dual‐layered Raman dye DTNB that functioned as SERS tags, which were responsible for the ultrasensitive and bioconjugation flexibility in the detection of antibody. Human IgM quantification was done by monitoring SERS signal on T‐line with the help of this SERS‐LFIA strip, and a very low limit of detection of 0.1 ng/ml was achieved. This SERS method was found to be 100 times more sensitive than quantified calorimetric method (Figure 8).^[^ ^154^ ^]^ In another study, a similar SERS‐based LFIA technique has been reported for the rapid and sensitive detection of *E. coli* O157:H7 in food sample. Specific monoclonal antibody (McAb) for *E. coli* O157:H7 was applied onto Au@Ag core–shell nanostructures, and loaded with dual‐layered Raman signal reporter DTNB, worked as SERS nanotags. Sensitive and accurate quantification of *E. coli* O157:H7 was done by monitoring Raman signal intensity at 1335 cm^−1^ peak on T‐line in a broad liner range of 10^1^–10^9^ CFU/ml combined with 6.94 × 10^1^ CFU/ml LOD.^[^ ^155^ ^]^

Similarly, the signal amplification of catalytic hairpin assembly (CHA) coupled with SERS‐LFA biosensor was proposed for the detection of two different lung cancer‐linked miRNA biomarkers including miR‐31‐5p and miR‐196a‐5p. In this study, biotin molecules present on the surface of Au–Ag NSs were exposed when two DNA hairpins self‐assembled themselves into a double‐stranded DNA molecule in the presence of the targeted miRNAs. Hence, the targeted miRNAs entered the next cycle, while SERS signal were strongly amplified by the concentration of SERS complexes on T1 and T2 lines. This biosensing platform provided fast, ultrasensitive, highly specific, and simultaneous detection of both lung cancer biomarkers: miR‐196a‐5p and miR‐31‐5p, with a LOD of 1.171 and 2.251 nM in phosphate buffer, and 1.681 and 2.603 nM in blood, respectively.^[^ ^156^ ^]^

Recently, a novel SERS‐LFA approach for the detection of chloramphenicol antibiotics (CAPs) has been published, in which core–shell Au@Ag NPs modified with an antibody were utilized for the fabrication of SERS nanotags. By using this strip based on above mentioned approach, the highly sensitive and efficient quantification analysis of CAP of 36 ng/ml, thiamphenicol (TAP) of 0.20 ng/ml, and florfenicol (FFC) of 0.78 ng/ml were achieved at their LOD.^[^ ^157^ ^]^

### 4.5 Detection of antibiotics, mycotoxcins, and aflotoxcins

#### 4.5.1 Detection of kanamycin in milk

Amino sugars are linked by a glycosidic bond to an aminocyclitol ring nucleus that forms a big class of antibiotics known as aminoglycosides. Aminoglycosides including kanamycin (KANA), neomycin, streptomycin, and gentamicin, etc., are widely used in agriculture and animals for protection against different pathogenic bacteria.^[^ ^158^ ^]^ Because of excessive and improper use, aminoglycosides remain active in animal‐origin foods that can result in antibiotic resistance and endanger public health.^[^ ^159^ ^]^ Among aminoglycosides, Kanamycin (KANA) is important as it is a broad‐spectrum antibiotic that can disrupt protein formation, and inhibit both gram‐positive and gram‐negative bacteria.^[^ ^160^ ^]^

When an animal is treated with Kanamycin for bovine mastitis, KANA may become a part of its milk, and then it can travel to human bodies via milk.^[^ ^161^ ^]^ The accumulation of KANA can lead to nephrotoxicity, ototoxicity, hepatotoxicity, and allergic reactions.^[^ ^162^ ^]^ Therefore, it is very important to detect KANA in animals derived from food sources, and for those reasons, DNAs probe was installed on the surface of the Au NPs that were later hybridized with KANA aptamer to form a SERS aptasensors, which were covered by Ag shells. In order to have a strong Raman signal and achieve low detection value in KANA, the aptamer was modified with the Raman reporter cyanine‐3 (Cy3).^[^ ^163^ ^]^ However, the Raman reporter Cy3 was unstable, and sensitive to light. Besides, the reported sensing principle was complex, and biosensor preparation time took more than a day. To counter all these flaws, a simple, rapid, and ultrasensitive SERS‐based aptasensor was presented that only needed less than 4 h for its synthesis. This aptasensor used 4‐mercaptobenzoic acid (4‐MBA) to modify Au@Ag NPs, and was optimized to show a value of 142 pg/ml for detecting KANA.^[^ ^164^ ^]^ Norfloxacin, another antibiotic is belonging to the Fluroquinolone family successfully detected in spiked fish muscles with the help of a SERS‐based Au@Ag nanosensor.^[^ ^165^ ^]^

#### 4.5.2 Detection of aflatoxin B1 in nuts and grains

**FIGURE 9:**
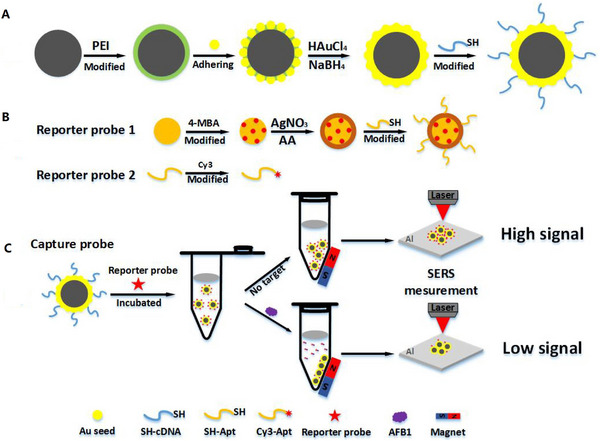
Schematic illustration of ultrasensitive determination of AFB1 based on combining multifunctional capture probes (Fe_3_O_4_@Au NFs‐cDNA) with strong Raman signals of reporter probes. (A) Functionalization of Fe_3_O_4_@Au NFs with Cdna. (B) Synthesis of reported probe 1 (Au‐4MBA@Ag NSs‐Apt) and reporter probe 2 (Cy3‐Apt). (C) SERS detection of AFB1 at different concentrations using the laser confocal Raman microscope. Reproduced with permission.^[^ ^170^ ^]^ Copyrights 2020, Elsevier

Aspergillus flavus and Aspergillus parasiticus can cause the production of Aflatoxins from a family of mycotoxins.^[^ ^166^ ^]^ Aflatoxins are primarily composed of aflatoxin G1 (AFG1), aflatoxin B2 (AFB2) and aflatoxin B1 (AFB1).^[^ ^167^ ^]^ Aflatoxin B1 is the most harmful contributor among these because of its high toxic nature and strong magnetic fields. For these particular reasons it is characterized as a group 1 carcinogen by the international agency for research on cancer.^[^ ^168^ ^]^ A number of food sources can be affected by the contamination of the Aflatoxin B1, mostly the plant based food sources such as corns, peanuts grains, etc.^[^ ^169^ ^]^ To detect the AFB1 proteins, a SERS aptasensor has been developed, which worked by combining the Au@Ag nanospheres with the Fe_3_O_4_@Au nanoflowers. To make an aptasensor with very high Raman signals, SH‐cDNA modified Fe_3_O_4_@Au NFs and SH‐Apt modified Au‐4MBA@Ag NSs were utilized for recognition. The LOD reached up to 0.4 pg/ml, and it could even detect the AFB1 at trace levels. It was found that triggering the release of Au‐4MBA@Ag NSs from Fe_3_O_4_@Au NFs could enhance the Raman signals (Figure 9).^[^ ^170^ ^]^

#### 4.5.3 Detection of ochratoxin A and zearalenone in plant‐based foods

Aspergillus and Penicillium can produce a toxin named ochratoxin A (OTA), and international agency for research on cancer (IARC) has put this chemical in an II‐B group of toxins that can cause cancer. These toxins can show properties like hepatoxic, teratogenic, etc.^[^ ^171^ ^]^ Different plant‐based foods including cereals, corn, coffee, and wine are a direct source of OTA toxicity.^[^ ^172^ ^]^ Another group III carcinogenic toxin known as zearalenone (ZEN) can cause reproductive toxicity, carcinogenicity, and neurotoxicity.^[^ ^173^ ^]^

**FIGURE 10:**
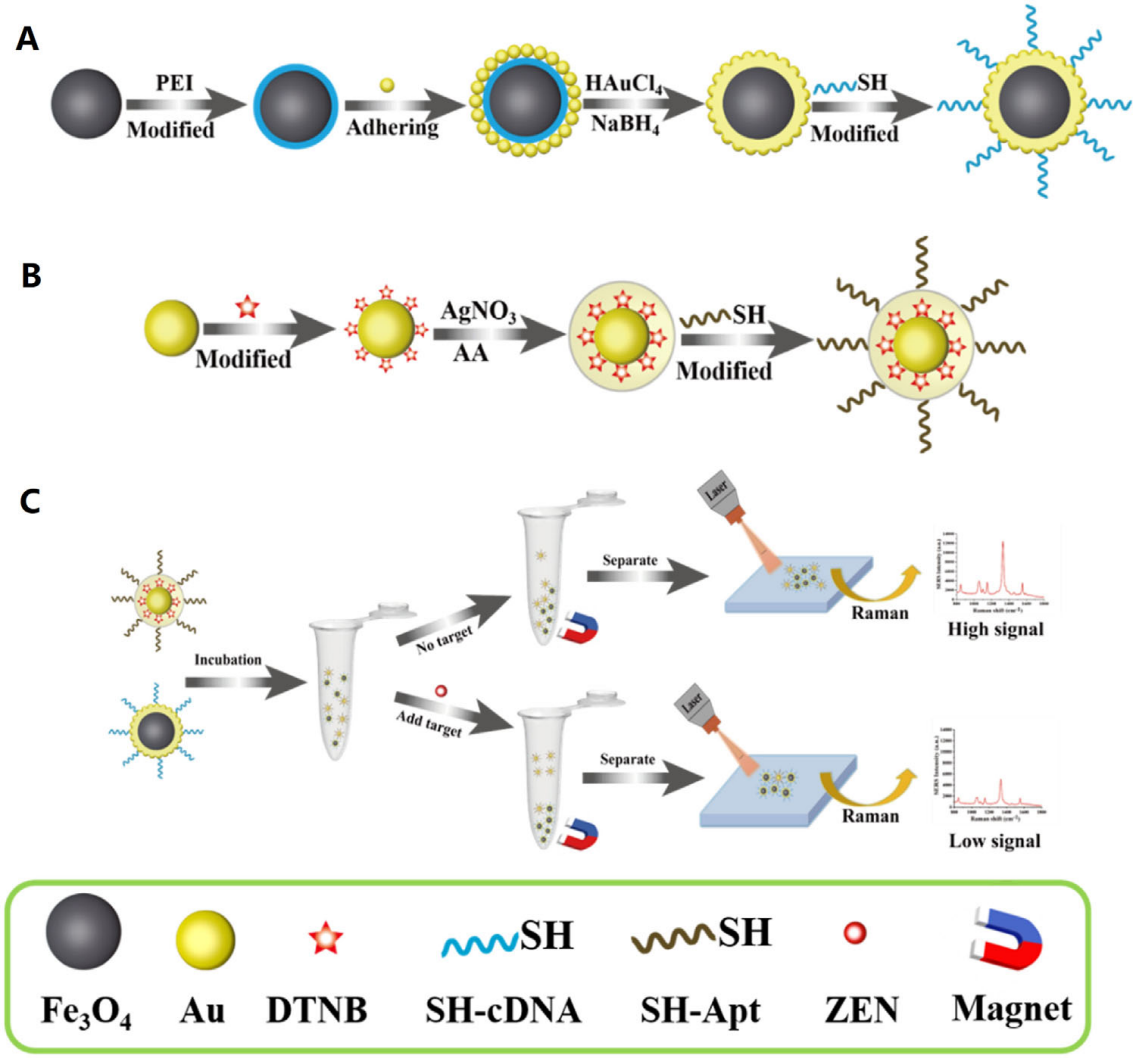
Schematic representation of the universal surface‐enhanced Raman scattering (SERS) aptasensor platform for trace detection. PEI, polyethyleneimine; AA, ascorbic acid. (A) Fabrication of monodispersed Fe_3_O_4_@Au MNPs and functionalization it with SH‐cDNA. (B) Synthesis of Au@DTNB@Ag CS and functionalization it with SH‐Apt. (C) Preparation of the SERS aptasensor for ZEN detection. Reproduced with permission.^[^ ^175^ ^]^ Copyrights 2021, Elsevier

*Fusarium* and *Gibberella* cause the production of ZEN that infects barley, wheat, and corn. Both these toxins impose a grave threat to the health of animals as well as humans, therefore, their detection is of utmost importance.^[^ ^174^ ^]^ A new hyper‐sensitive SERS aptasensor has been reported, which was composed of Fe_3_O_4_@Au MNPs combined with Au@5,5 dithiobis‐(2‐nitrobenzoic acid) (DTNB)@Ag CSs, and had a LOD of 0.001 ng/ml^[^ ^175^ ^]^ (Figure 10). A Fe_3_O_4_@Au magnetic nanoparticles (MGNPs) and Au@Ag nanoprobes were modified with the Raman reporter 5,5‐dithiobis (2‐nitrobenzoic acid; DTNB). The designed Au‐DTNB@Ag NPs‐based SERS aptasensor was used for the detection of OTA, which had a LOD of 0.48 pg/ml.^[^ ^176^ ^]^

In order to continuously detect ZEN and OTA through utilizing SH‐cDNA modified gold nanorods, a new SERS‐based aptasensor was reported. This method was not only capable of detecting ZEN and OTA, but it also distinguished between the two mycotoxins. Furthermore, multiple types of mycotoxins could be detected at the same time by changing the aptamer and signal probes. A LOD of 0.054 ng/ml was reported for the detection of ZEN and OTA by this unique and novel approach.^[^ ^177^ ^]^

### 4.6 SERS‐based immunoassays

Recently, SERS‐based immunoassay approaches were reported with a sandwiched immune structure, which commonly consisted of an immune probe, an antigen target, and a SERS‐active substrate. The excellent optical characteristics of immune substrate and immune probe performed a key role in achieving ultrasensitive detection of tumor biomarkers. SERS‐active substrate and probe were generally prepared by Au and Ag NPs that showed outstanding SERS properties due to their exceptional LSPR effect.^[^ ^178^ ^]^ Important cancer biomarkers like prostate‐specific antigen, carcinoembryonic antigen (CEA), mucoprotein1 (mucin‐1), and alpha‐fetoprotein (AFP) were detected successfully by using SERS‐based immunoassays.^[^ ^179^ ^]^ α‐Fetoprotein or AFP was not only a key biomarker for the diagnosis of hepatocellular carcinoma but was also found to be vital in hepatectomy, chemotherapy, and liver transplantation prognosis. According to clinical studies, a patient who was on liver transplant waiting list could experience considerably worse as his baseline AFP level would reach 4200 ng/ml, and there would be a steady increase of 415 ng/ml per month in his AFP level.^[^ ^180^ ^]^ Therefore, for highly sensitive detection of AFP, a SERS‐based immunoassay complex had been designed by using Au–Ag NPs with good monodispersity, and in there, Ag‐AgBr hybrid NSs provided great stability. Consequently, a broad linear range of 2 fg/ml–0.8 g/ml and a LOD as low as 1.86 fg/ml was achieved by detecting AFP in human serum using this SERS‐based immunoassay.^[^ ^181^ ^]^

Another approach was proposed by using antibody conjugated SiO_2_ coated Au/Ag core–shell nanostars (AuNS@Ag@SiO_2_) as SERS probes, and a nitrocellulose membrane labeled with an antibody was utilized to selectively capture AFP. 4‐MBA was used as a Raman reporter, and an LOD of 0.72 pg/ml was achieved for ultrasensitive detection of AFP.^[^ ^182^ ^]^ Similarly, a novel SERS‐based immunoassay was reported for fast, ultrasensitive, and selective detection of AFP. This platform used Au nanospheres coated with Ag (Au@Ag), a very thin SiO_2_ shell, and AuNP satellites, which provided high coverage and formed a multilayered plasmonic core–shell nanostructure. The Au@Ag core provided a remarkable SERS activity, and the SERS signal was further enhanced by the broad range plasmon coupling of Au@Ag with AuNP via SiO_2_ layer. High biocompatibility and stability were achieved by the outer AuNP satellites. Thus, a broad linear range of 1 fg/ml to 1 ng/ml and a very low LOD of 0.3 fg/ml for AFP detection was achieved by using this novel SERS‐based sandwiched immunoassay.^[^ ^183^ ^]^

**FIGURE 11:**
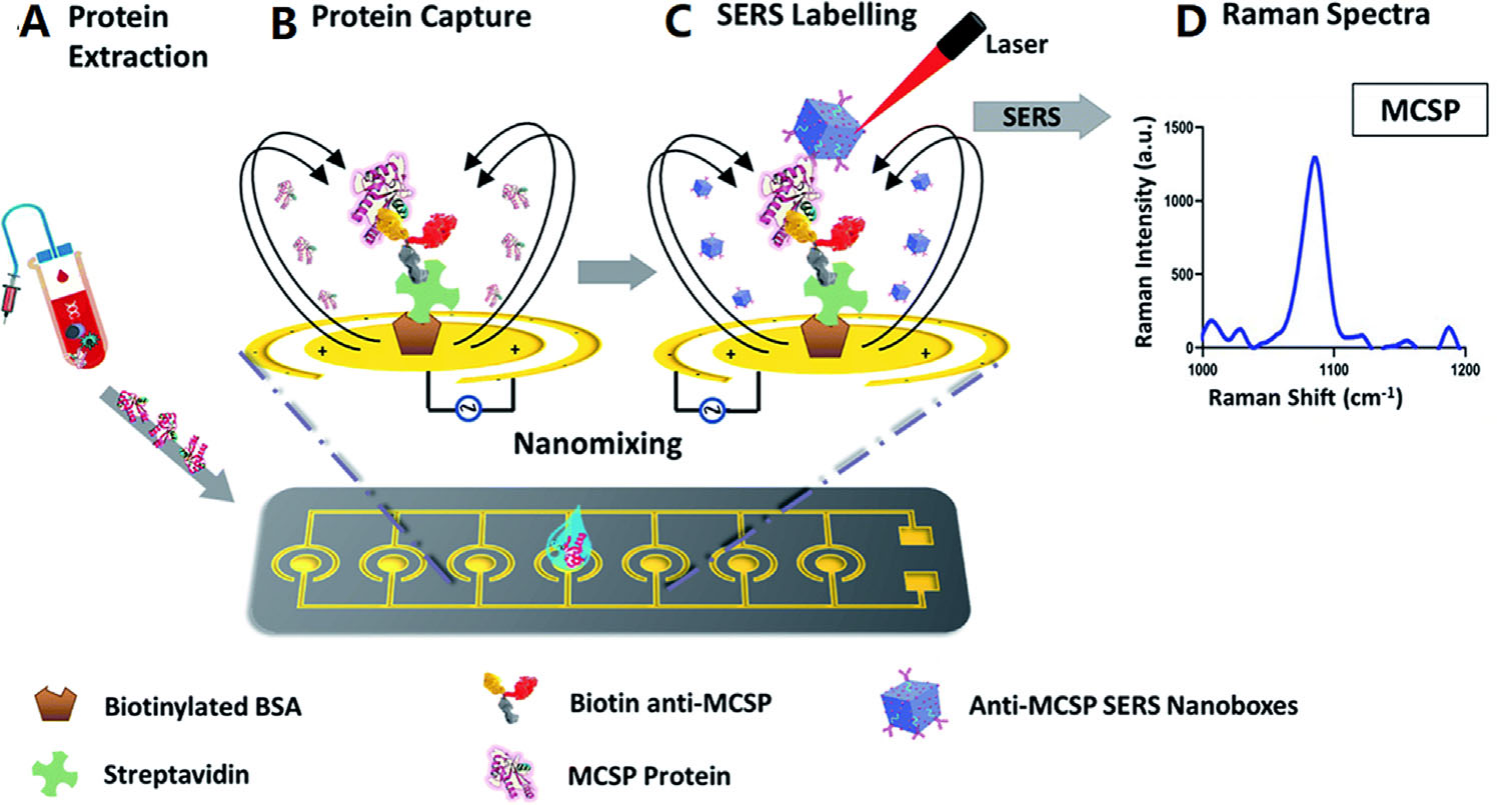
The methodological approach for melanoma biomarker detection using microchip‐SERS platform. (A) Extraction of protein lysate and immunoprecipitation of pure MCSP protein. (B) ac‐EHD induced nanofluidic mixing for specific MCSP protein capture. (C) SERS labeling of target MCSP with the anti‐MCSP conjugated SERS nanotags with ac‐EHD nanomixing. (D) The molecules are excited with laser for Raman scattering and a characteristic peak is obtained at 1075 cm1, peak height corresponds to the concentration of the target antigen. Reproduced under the terms of the CC BY‐NC 3.0 license.^[^ ^184^ ^]^ Copyright 2020, Aswin Raj Kumar et al.

According to various studies, a possible noninvasive technique for early diagnostics and therapeutic management of cancer is the detection of circulating biomarkers in liquid biopsies. Melanoma chondroitin sulfate proteoglycan (MCSP) is one of those circulating biomarkers. MCSP is a membrane protein that is involved in melanoma cell migration and invasion of nearby tissues. Therefore, its soluble form (sMCSP) could be a potential cancer diagnostic biomarker, and it is very difficult to selectively detect sMCSP in complex body fluids due to its low concentration in early stages of disease. Recently, a high‐throughput and ultra‐sensitive microchip based on SERS immunoassay was reported for simultaneous detection of as many as 28 samples (Figure 11). The simulation of AC‐induced nanofluidic mixing to improve target‐sensor collisions was the key for rapid and ultrasensitive sMCSP detection with a low LOD of 0.79 pM (200 pg/ml).^[^ ^184^ ^]^

Similarly, dual‐SERS tag sandwich assays were reported to detect interleukin 6 (IL‐6), which was a pleiotropic cytokine responsible for hematopoiesis, normal immune function, and metabolism. The normal value of IL‐6 concentration in circulating fluid was 1 pg/ml, during chronic inflammation and autoimmune disorders, this concentration of IL‐6 elevated up to > µg/ml. Therefore, SERS‐active metal nanoparticles provided essential enhancement to this SERS‐based immunoassay for detection of IL‐6, and a broad linear range of 0–1000 pg/ml with LOD as low as 25.2 pg/ml^‐1^ (RSD < 10%) was achieved.^[^ ^185^ ^]^

### 4.7 Detection of heavy metals

Lead (Pb) is a toxic heavy metal that may be found in soil water due to leaching process from hazardous industrial wastes. Lead ion (Pb^2+^) has imposed serious environmental and health threats to human beings.^[^ ^186^ ^]^ For living organisms, long term exposure to Pb^2+^ leads to its aggregation in their bodies. It has been related to lethal diseases including neurotoxicity, hepatotoxicity, and nephrotoxicity even at very low concentration.^[^ ^187^ ^]^ Pb^2+^ may enter human body via different mediums including water, air, and various food‐based sources. According to US Environmental Protection Agency (USEPA), the maximum limit of Pb^2+^ intake in drinkable water is about 15 ppb (72 nM), and in human serum is about 100 ppb (483 nM).^[^ ^188^ ^]^ Therefore, quantitative detection is very important, and for this purpose, a DNAzyme‐based SERS biosensor had been reported with Fe_3_O_4_@Au@Ag NPs as SERS substrate and Cy3‐labeled thiolated DNA as SERS probe. As Pb^2+^ was bound, the Cy3‐labeled thiolated DNA was released by the Pb^2+^‐dependent DNAzyme, and the decrease in SERS intensity was observed. The LOD for detecting Pb^2+^ by using this process was as low as 5 pM.^[^ ^189^ ^]^ Another reusable and highly specific SERS biosensor for Pb^2+^ detection was reported where SERS substrate was combined with graphene monolayer, Au–Ag based perforated GaN, and Cy3‐DNAzyme was used as probe. With its dual enhancement capability, this SERS biosensor exhibited LOD of 4.3 pM with an extensive linear detection range of 0.01–100 nm.^[^ ^190^ ^]^

**FIGURE 12:**
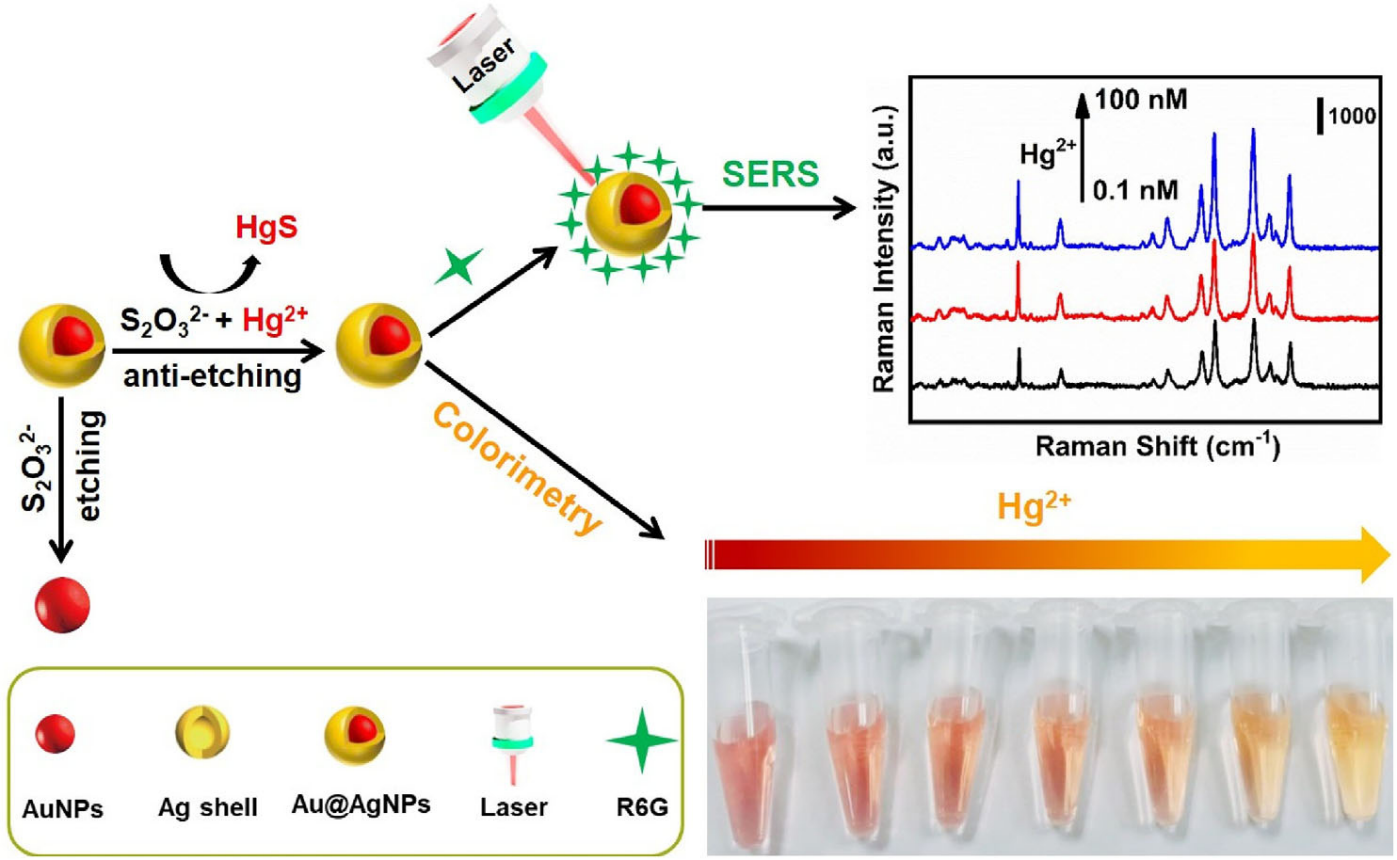
Principle diagram of the colorimetric and SERS dual‐mode probe for determination of Hg^2+^ based on controllable etching unmodified Au@Ag NPs. Reproduced with permission.^[^ ^196^ ^]^ Copyright 2021, Elsevier

Highly toxic heavy metal Mercury (Hg) raises serious environmental issues and it is released from various processes including volcanic eruption, combustion of fossil fuel, coal mining, and solid waste incineration.^[^ ^191^ ^]^ Excessive exposure to Hg^2+^ results in its accumulation in human and animal bodies. Hg^2+^ enters human body through food chain and is responsible for causing serious life‐threatening diseases in humans including renal failure and neurodegeneration.^[^ ^192^ ^]^ The 0.02 mg/kg is the minimal limit of Hg^2+^, and anything more than this standard may result in serious consequences.^[^ ^193^ ^]^ So, the highly sensitive and rapid detection of Hg^2+^ has been an urgent need of time. For this purpose, Raman biosensor with cascade sensitivity was fabricated via parallel assemblies of Au@gap@AuAg NRs with 4‐NTP as Raman reporter. This Raman biosensor achieved intransitivity for Hg^2+^ detection with LOD of 1 pg/ml.^[^ ^194^ ^]^ Similarly, another versatile dual‐channel Au modified with Ag/graphene (Au@Ag‐G) biosensor was reported for Hg^2+^ detection that readout simultaneously both SERS and fluorescence signals. Magnetite colloid nano clusters/polymethacrylic acid magnetic beads (MCNCs/PMAA MBs) were incorporated for specifically capturing Hg^2+^ ions. By this novel approach, a LOD of 0.33 ppb was successfully accomplished.^[^ ^195^ ^]^ Based on the etching of Au@Ag NPs, a colorimetric and SERS bimodal detection of Hg^2+^ was reported, and this unique approach reached LOD of 0.1 nm^[^ ^196^ ^]^ (Figure 12).

### 4.8 Detection of biomolecules

Phosmet, permethrin, carbaryl, and cypermethrin are typical pesticides used in fruit and vegetable cultivation and postharvest treatments. Pesticide residual contamination in soil, environment, and food has sparked widespread fear, as it causes serious health hazards to human beings due to its highly toxic nature. Adulteration of pesticides residues in agricultural products is critical issue for food safety regulations. Current approaches for the detection of pesticides traces in food are unreliable, ineffective, and time consuming. Research community is working hard to devise a simple, rapid, and effective method for trace pesticide analysis. In this regard, Sivashanmugan and his team employed nano‐indentation and focus‐ion beam method to fabricate Au@Ag@Au nanorods and gold nano‐cavity array to detect various pesticide residues. Furthermore, Au@Ag@Au‐NRs probes efficiently detected several pesticide residues even at low concentrations.^[^ ^197^ ^]^

Bisphenol A (BPA) is an unsteady lipophilic compound that is mostly used in the manufacturing of food containers, baby bottles, crockery, and medical equipment. Citizens are constantly exposed to BPA due to effluents discharged from the plastic manufacturing sector. BPA is an endocrine disrupting agent that has the potential to harm human health even at minute concentrations. By binding to estrogen receptors, it harms the endocrine glands of humans, leading to the lack in immune function, which forms cancer and poor reproduction. To address these issues, SERS‐based aptamer sensor for observing the Bisphenol A (BPA) was constructed. Chitosan topped four‐mercaptobenzoic acid (4‐MBA) labeled Au@Ag nanoparticles were developed to detect BPA. The charge differences between the nucleic acid and noble Au/Ag metals lead to the improvement of Raman detection capacity. This SERS based nano‐sensor provided a limit of recognition 2.8 picogram/ml in the buffer solution, and a concentration ranged from 0.01 to 1 ng/ml. These results revealed the ultra‐sensitivity of this SERS technique, which had 2–3 levels lower magnitude than the other available BPA detection approach, including HPLC, and mass spectrometry.^[^ ^198^ ^]^

Glutathione (γ‐Lglutamyl‐L‐cysteinyl‐glycine, GSH) is the most abundant endogenous thiol composite, having in vivo concentration with range of 0.5 to ∼ 10 mM. Furthermore, several lethal diseases like Parkinson's, AIDS, cystic fibrosis, and cancer diseases, all are linked to GSH in its reduced form (95%). The efficacy of anticancer therapy is also reported to be hampered by an elevated level of GSH. Recently, an extremely sensitive and selective Au–Ag NBs/Nc‐CuII nanobowls was fabricated for the sensitive and targeted detection of GSH. The redox reaction‐based conversion of Nc‐CuII into Nc‐CuI in the existence of GSH resulted in the alterations of SERS spectra. Thus, it revealed a great prospective for diagnosis and prediction of diseases related with the GSH concentration.^[^ ^199^ ^]^

Triazole such as paclobutrazol is an important plant growth regulator, which is involved in retarding the plant vegetative growth and increasing crop production. This biochemical is applied in agricultural industry due to its great efficacy and lower toxicity to crop plants. It has a broad‐spectrum agronomic impact on crops and ornamental plants. As it retards the plant growth, plant inter‐nodal distance reduces which ultimately reduces the lodging risk. However, their widespread use has created a serious threat for both human health and environment safety. Their residues in soil have been linked to human carcinogenicity and genotoxicity. For the food biosafety measurements, nanosensor was prepared to detect the trace contamination of paclobutrazol in the soil and end‐use food. Kou and his team developed molecular imprinted polymer‐based SERS biosensor for accurate, targeted, and quantitative evaluation of paclobutrazol traces in food and soil. In this approach, FSAA nanocomposites (Fe_3_O_4_/SiO_2_‐Au/Ag) alongside tunable Au and Ag inter‐particle apertures were fabricated. After modification in the Au–Ag nanogaps, and evaluating the SERS detection improvement, the ideal SERS substrate‐FSAA‐40 was developed. In combination of FSAA‐40 and MIPs, paclobutrazol was selectively detected with low limit of recognition 0.075 µg/g in food and soil, even lower concentration detection of paclobutrazol as 0.075 to 12.75 µg/g.^[^ ^200^ ^]^

The separation of molecules from the complex mixtures, and assessment of pre‐concentration of molecules in the mixtures are difficult tasks. For this purpose, SERS probe‐based nanosensor was fabricated for the accurate separation and ultra‐sensitive evaluation. Recently, plasmonic filters were synthesized from MoS_2_@Au/Ag nanostructures, and SERS probe‐based assessment was utilized in complex molecules mixtures.^[^ ^201^ ^]^ Most importantly, interaction of glass fiber and SERS‐based nanostructures ensured manageable separation and preconcentration of molecules in the complex mixture that made the selective detection possible for each molecule in the mixture. For the detection of crystal violet molecules, Au/Ag complex of micro/nanostructures was prepared by employing double pass porous alumina template assisted vacuum thermal evaporation technology.^[^ ^202^ ^]^ The resultant cascade, containing the multiple ultra‐micro nanogaps, was applied as a SERS substrate for the assessment of crystal violet molecules.

Novel multifunctional nanosensing technique based on graphene like titanium carbide (Ti_2_C MXene)/gold‐silver nano‐shuttles (NSs) was successfully designed. This platform employed both SERS substrate and electrochemical intelligent analysis to observe the ultra‐trace carbendazim (CBZ) contaminations in tea and rice.^[^ ^203^ ^]^ Ti_2_C MXene nanosensor was manufactured by selectively fabricating the Al films of titanium carbide with the hydrofluoric acid at elevated temperature calcination. The synthesis of Ti_2_C MXene/Au–Ag NSs was done under dark environment with the feature of coarse surface, improved conductivity, extraordinary stability, and substantial SERS signals enhancement. The machine learning for the detection of CBZ traces was evaluated through multiple algorithms including synthetic neural network, relevance vector machine (RVM), and support vector machine. RVM showed better performance than other algorithms for the detection of CBZ residues. This RVM demonstrated the good LOD as 0.002 µM, and the SERS nanoshuttles detected the CBZ traces with LOD of 0.01 µM.

A novel SERS‐based strategy was developed for the spatiotemporal and conservative evaluation of extracellular metabolites in the microbial populations. In this approach, special nanoprobes consisting of mSiO_2_ nanorattles containing single core–shell Au@Ag NPs (Au@Ag@mSiO_2_) were fabricated. Initially, exudation of the microbial metabolites and dynamic interaction among the microbial communities were detected with powerful SERS‐based nanosensor.^[^ ^204^ ^]^ According to literature, one of the most common allergies in drug utilization was Penicillin allergy with a range of 8 to 25%.^[^ ^205^ ^]^ One of the major causes of allergy was the Penicilloyl protein (P‐protein), which is derived after the rupture of β‐lactam ring from penicillin. According to a study, P‐protein could potentially cause an onset allergic reaction.^[^ ^206^ ^]^ Prior studies have revealed that P‐protein conjugates could cause anaphylactic reactions even at very low concentration.^[^ ^207^ ^]^ It was learned that one of the best ways to evaluate an allergic reaction was through the detection of P‐protein in the blood that is found in huge amount in allergic reactions.^[^ ^208^ ^]^

**FIGURE 13:**
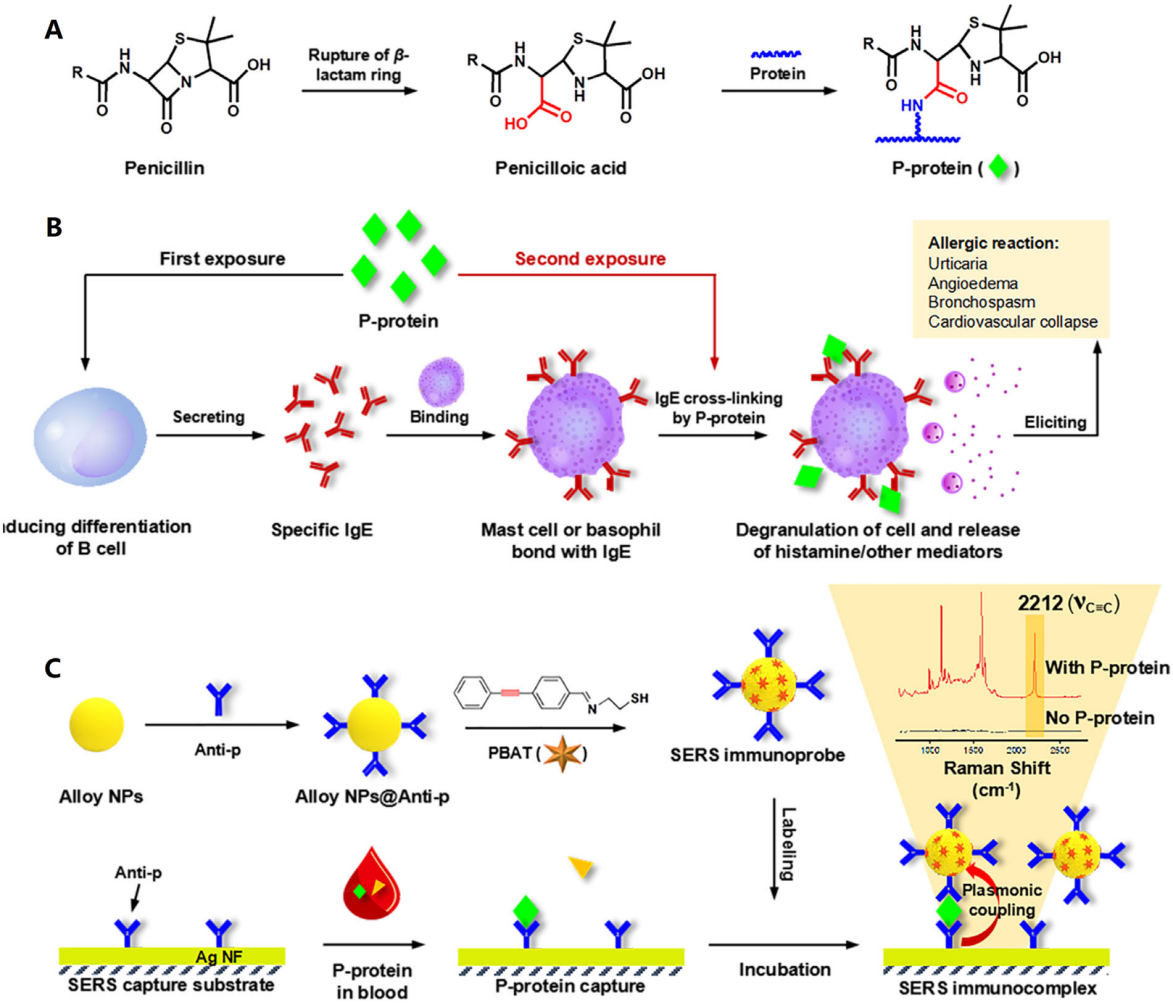
Schematic diagrams of (A) the formation of major allergenic determinant (penicilloyl) and P protein from penicillin drugs, (B) the mechanism of antibody‐mediated hypersensitivity reaction, and (C) the alkyne response‐based SERS immunoassay for P‐protein. Reproduced with permission.^[^ ^209^ ^]^ Copyright 2021, Elsevier

A new report has been presented where a SERS sensor composed of Ag–Au alloy nanoparticles was able to detect P‐protein. This proposed study was not only able to enhance the sensitive Raman response with 2212 cm^−1^ peak in the silent region, but also prevent the fluorescence effect that could come from the biological samples. Besides, they were able to get very specific identification of P‐protein from the complex structures. The best achievement was that it could not only decrease the time of detection, but also made it more accurate, feasible, and trustworthy detection of allergens in the blood. In addition, This SERS sensor reached a very low LOD (0.329 pg/ml)^[^ ^209^ ^]^ (Figure 13).

### 4.9 miRNA detection

Micro‐RNA (miRNA) is composed of 20 to 25 nucleotides, which is present in cells, and several of them are circulating in serum. miRNA plays an important role in post transcriptional processes during the expression of gene. Among them, many miRNAs function as oncogenes are served as tumor suppressors. Recent studies reported that higher expression level of miRNA‐141 is found in breast cancer patient's blood. Similarly, miRNA‐21 can be found in serum of patients, who have cardiovascular diseases and pulmonary diseases. Hence, miRNAs are reliable biomarkers for early detection of cancer.^[^ ^210^ ^]^

Recent research has been based on Au@Ag core–shell nanorods (Au@Ag NRs) SERS sensor and cascade DNAzyme amplifier (CSA) to detect microRNA‐21.^[^ ^211^ ^]^ Au nanowires @Ag nanoparticles material used as SERS biosensor with multiple signal amplifications were reported to detect miRNA‐41.^[^ ^212^ ^]^ Multiple signal amplification has been achieved by sharp tips of nanowires. Ag staining over the Au nanowires (as Ag increase SERS enhancement), and a DNA Skelton were spread through hybridization chain reaction (HCR), which entrapped more Ag while depositing, and increased the surface area for Raman reporter R6G. On the other hand, capture unit was prepared by attaching capture probe on the Fe_3_O_4_@AuNPs.

**FIGURE 14:**
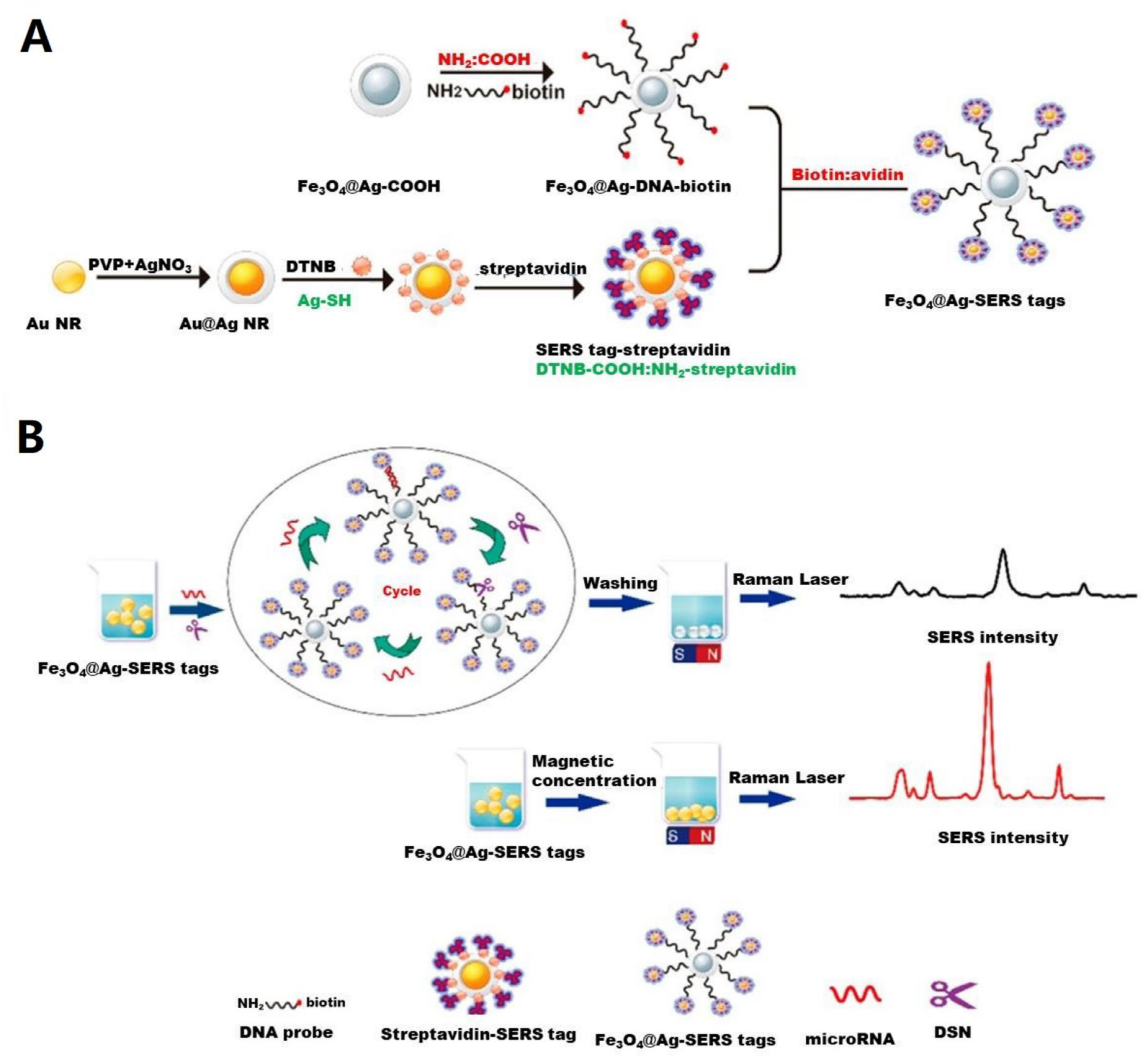
Schematic illustration of microRNA SERS detection based on DSN‐assisted target recycling signal amplification. (A) The synthesis process of Fe_3_O_4_@Ag‐SERS tags. (B) DSN‐assisted SERS detection of microRNA. Reproduced with permission.^[^ ^213^ ^]^ Copyright 2019, Elsevier

Another biomarker, miRNA‐10b has been enlisted for the detection of pancreatic cancer. This miRNA was quite specific that it could distinguish between chronic pancreatitis and pancreatic cancer. A SERS biosensor based on Au@Ag bimetallic alloy for the detection of miRNA‐10b from the blood serum residual and exosomes have been reported.^[^ ^213^ ^]^ The biosensor was comprised of two parts: Fe_3_O_4_ @Ag‐DNA and SERS tag Au–Ag–DTNB. SERS tag was attached with the Fe_3_O_4_ @Ag‐DNA, which released in the presence of miRNA biomarker, and SERS intensity quenching could be triggered. The recorded LOD for this dual SERS biosensor was 1 aM (Figure 14).

Another important biomarker miRNA‐21 played a vital role in development of many diseases (Oncogenesis, cardiovascular diseases). There are multiple SERS‐based biosensors that were designed in the recent years to detect miRNA‐21, and each method had its own importance and pathways. Most of these biosensors used Au@Ag bimetallic alloy as SERS tag. A SERS‐based sandwich biosensor was designed following the principle of complementary base pairing of DNA. Au/Ag‐4MBA served as probe, and silver NPs decorated on titanium oxide nanowires (Ag@TiO_2_) served as substrate. Au/Ag‐4MBA/5´‐NH_2_‐ssDNA probe was constructed by adding 5´‐NH_2_‐ssDNA to 4‐MBA and Ag/TiO_2_‐3´‐NH_2_‐ssDNA. Hence, a sandwich model was established in the presence of miRNA‐21, which bound probe and single stranded DNA, thus, enhanced the SERS signals. Reported LOD for this SERE‐based bioassay was 0.75 fM.^[^ ^214^ ^]^

Similarly, another efficient SERS based biosensor based on lateral flow principle was synthesized by coupling SERS substrate with catalytic hairpin assembly (CHA). 4‐MBA was imbedded in Au@Ag core@shell SERS probe. This SERS biosensor gave LOD as low as 84 fM.^[^ ^215^ ^]^ Another SERS‐based biosensor was synthesized to detect miRNA‐21 using magnetic NPs Fe_3_O_4_ and SERS substrate Au@Ag core@shell NPs.^[^ ^216^ ^]^ Its SERS coupled the duplex specific nuclease signal amplification (DSNSA)‐based miRNA‐21 biosensor. SERS probe was designed by DTNB attached in between the Au core and Ag shell NPs, resulting in Au‐DTNB‐Ag NPs (ADANPs). In the next step, a capture probe (CP) linked with ADA NPs (CP‐ADA NPs), was covalently bonded with Fe_3_O_4_ to form a sandwich on the end of ADA‐NPs, and other Fe_3_O_4_ attached to 3′ and 5′‐end of CP. In the presence of miRNA‐21, it was linked with the CP and a hybridized RNA/DNA heteroduplex emerged. On using DSN, this heteroduplex split into small fragments, and resulted in release of ADA from the surface of Fe_3_O_4_. The release of ADA from Fe_3_O_4_ affected the SERS signals and thus, miRNA could be detected. The LOD of this biosensor was 0.084 fM.

Biomarker for breast cancer miR‐K12‐5‐5p was also detected using Au@Ag@porous GaN gallium nitride hybrid SERS substrate with R6G molecule. The LOD by this biosensor was recorded at 8.84 × 10^−10^ M.^[^ ^217^ ^]^ A number of methods have been reported for the detection of single miRNA, but very rare numbers of strategies have been reported to detect multiple miRNA.^[^ ^218^ ^]^ A catalytic hairpin self‐assembly (CHA) based‐SERS biosensor was reported to detect the multiple miRNAs at same time mixed in a single sample. Biosensor was based on two parts, first SERS tag, which was Au@Ag NPs coated with 4‐mercaptobenzonitrile (MPBN) as a Raman reporter, and a hairpin‐structured DNA sequence 2 (hp2). The second part was a sensor array with 4 different types of sensing units immobilized by four different (hairpin‐structured DNA sequence 1 (hp1). In the presence of specific miRNA, Au@Ag‐based SERS tag was captured with SERS sensing unit through a repeated specific catalytic hairpin self‐assembly (CHA) reaction. Due to the interaction between Au@Ag‐based SERS tag and Au/Ag‐NP layer, new hotspots were generated, which enhanced the SERS signals. Recent advances in the field of Au@Ag NPs SERS biosensorshave made significant strides in the past few years. This biosensors have shown promising results in detecting a wide range of biomolecules with high sensitivity and specificity. The advantages in Au@Ag NPs SERS biosensors from 2018 to 2022 are listed in the below Table 1. Table 1 presents a comprehensive overview of the recent developments in Au@Ag SERS biosensors, highlighting key parameters such as the LOD, linear range, and specific Raman reporters used. These data points are critical in assessing the performance of these biosensors and understanding their potential applications in various fields. These advances demonstrate the potential for Au@Ag NPs SERS biosensors to play a critical role in the early detection and diagnosis of diseases.

**TABLE 1:** Recent advances on developing Au@Ag NPs SERS Biosensors (2018–2022)

Analyte	Nanoprobe	Raman reporter	Linear range	LOD	Ref.
Lead ions (Pb^2+^)	Fe_3_O_4_@Au@Ag NPs	Cy3	‐	5 pm	^[^ ^189^ ^]^
Bisphenol A	4‐MBA coated Au@Ag NPs	4‐MBA	10 pM–100 nM	4.31 pM	^[^ ^198^ ^]^
miRNA‐141	AuNWs/HCR@AgNPs Fe_3_O_4_@AuNPs	R6G	‐	0.03 fM	^[^ ^212^ ^]^
Carbendazim (CBZ)	Ti_2_C MXene/Au–Ag NSs	‐	0.033–10 µM	0.01 µM	^[^ ^203^ ^]^
microRNA‐21	Au@4‐MBA@Ag NPs	4‐MBA	‐	84 fM	^[^ ^215^ ^]^
Mercury ion (Hg^2+^)	Au@gap@AuAg NRs	4‐NTP	‐	0.001 ng/ml	^[^ ^194^ ^]^
Aflatoxin B1 (AFB1)	SH‐cDNA modified Fe_3_O_4_@Au nanoflowers and SH‐Apt modified Au@Ag nanospheres	Cy3	0.000–100 ng/ml	0.40 pg/ml	^[^ ^170^ ^]^
miRNA (miR‐133a)	Hollow Au/Ag SERS Nanoprobes	4‐NBT	‐	0.306 fM	^[^ ^219^ ^]^
Uric acid (UA) and ascorbic acid (AA)	Trisoctahedral Au core‐silver Ag shell nanocubes	4‐MBA	‐	0.36 µM for UA and 0.019 µM for AA	^[^ ^220^ ^]^
ZEN	Fe_3_O_4_@Au and Au@Ag core–shell NPs	‐	0.005–500 ng/ml	0.001 ng/ml	^[^ ^175^ ^]^
Prostate cancer biomarkers	Au@AgNRs	4‐MBA	‐	0.94 fg/ml	^[^ ^180^ ^]^
Mercury ion (Hg^2+^)	Au@Ag‐GU	4‐MBA	‐	0.33 and 1 ppb	^[^ ^195^ ^]^
*E. coli*, *S. aureus*, and *Salmonella*	Magnetic Au@Ag NPs (MAu@Ag@MPBA)	4‐MPBA	‐	‐	^[^ ^134^ ^]^
microRNA‐10b	Fe_3_O_4_ @Ag‐DNAAu@Ag@DTNB	DTNB	‐	1 aM	^[^ ^213^ ^]^
Alpha‐fetoprotein (AFP)	Au@Ag@SiO2‐AuNP	4‐MBA	1 fg/ml–1 ng/ml	0.3 fg/ml	^[^ ^183^ ^]^
CD63, HER2 and EpCAM	Au@Ag NCs on Au FON	R6G	‐	50 exosomes/ml	^[^ ^148^ ^]^
Lead ions (Pb^2+^)	Gr–Au–Ag–porous GaN	Cy3	‐	4.31 pM	^[^ ^190^ ^]^
*E. coli*, *S. aureus*, and *P. aeruginosa*	Au@AgNPs	R6G	‐	1 × 10^−9^ M	^[^ ^128^ ^]^
Ochratoxin A (OTA)	Au@DTNB@Ag	DTNB	‐	0.48 pg/ml	^[^ ^176^ ^]^
AFP antigen	Au–Ag alloy, Ag/AgBr NSs	4‐MBA	2 fg/ml–0.8 g/ml	1.86 fg/ml	^[^ ^181^ ^]^
Thiram (pesticide)	Ag@T‐A@SiO_2_@Au	4‐MBA	10^3^–10^8^ CFU/ml	10^3^ CFU/ml	^[^ ^221^ ^]^
EpCAM, ErbB2, and CD44	Ag‐encapsulated Au (Ag–Au)	DTDC, MGITC, and RBITC	‐	‐	^[^ ^147^ ^]^
SKBR3, HeLa, Jurkat T, LNCaP	MB‐Au@Ag NRs‐SiO_2_	DTNB, 4MBA	‐	‐	^[^ ^222^ ^]^
B16F10 ELVs	Au@AgNPs	‐	‐	‐	^[^ ^149^ ^]^
SERS Imaging	EGFR‐Au@Ag NR nanotags	DTNB	‐	‐	^[^ ^223^ ^]^
Paclobutrazol	Fe_3_O_4_@SiO_2_‐Au@Ag (FSAA)	‐	0.075–12.75 µg/g	0.075 µg/g	^[^ ^200^ ^]^
miR‐196a	AgNW@AuNPs	NBA, 5‐FAM	1 fM–100 pM	96.58 aM in PBS, and 130 aM in serum	^[^ ^224^ ^]^
Soluble sMCSP	Au–Ag alloy nanoboxes	4‐MBA	‐	0.79 pM	^[^ ^184^ ^]^
Ochratoxin A (OTA) and ZEN	Au NRs‐cDNA, Au@4‐MBA@Ag CS‐OTA and Au@DTNB@Ag CS‐DTNB aptamers	4‐MBA, DTNB	0.01–100 ng/ml	0.018 ng/ml	^[^ ^177^ ^]^
α‐fetoprotein	AuNS@Ag@SiO_2_ nanostars	4‐MBA	3 pg/ml–3 mg/ml	0.72 pg/ml	^[^ ^182^ ^]^
Benzaldehyde and 3‐Ethylbenzaldehyde	MOF, ZIF‐8, Au@Ag nanocubes	‐	‐	1 ppb	^[^ ^225^ ^]^
Penicilloyl protein (P‐protein)	Au–Ag alloy nanoparticles	4‐MBA	‐	0.329 pg/ml	^[^ ^209^ ^]^
Pyocyanin	Au@Ag@mSiO_2_ nanorattles	4‐MBA	‐	‐	^[^ ^204^ ^]^
Interleukin 6 (IL‐6)	Au–Ag, Au–Au, and Ag–Ag	4‐MBA	0–1000 pg/ml	25.2 pg ml	^[^ ^185^ ^]^
Lactic acid (LA)	SFF‐Au/Ag nanodendrites	CV, 4‐MBA	10–50 mM	10^− 9^ M	^[^ ^226^ ^]^
Thrombin and PDGF‐BB	Au–Ag HNPs@DTNB and Au–Ag HNPs@NBA	DTNB, NBA	‐	4.837 pg/ml for thrombin and 3.802 pg/ml for PGDF‐BB	^[^ ^227^ ^]^
R6G, and FITC	MoS2@Au/Ag hybrids	‐	‐	10^−11^ M for R6G and 10^−12^ M for FITC	^[^ ^201^ ^]^
Mycoplasma pneumoniae (MP)	Au/DTNB@Ag/DTNB	DTNB	‐	0.1 ng/ml for human IgM	^[^ ^154^ ^]^
R6G, malachite green (MG), and uric acid (UA)	Au@Ag nanoislands	‐	0.01–100 µM for R6G, 0.001–1 µM for MG, and 100−500 µM for UA	‐	^[^ ^54^ ^]^
Permethrin, cypermethrin, carbaryl, and phosmet	Au/Ag/Au nanorods (NRs)	R6G	‐	10^−8^ M	^[^ ^197^ ^]^
Kanamycin	Au@Ag NPs	4‐MBA	6.67 × 10^−10^ g/ml – 2 × 10^−7^ g/ml	142 pg/ml	^[^ ^164^ ^]^
Kanamycin	Au@Ag NPs	Cy3	10 µg/ml–100 ng/ml	0.90 pg/ml	^[^ ^163^ ^]^
SERS imaging of Epithelial Cells	Au@polyDOPA@Ag	MB	‐	‐	^[^ ^228^ ^]^
Crystal violet (CV)	Au nano‐islands @ Ag‐frustum arrays	‐	‐	10^−10^ M	^[^ ^202^ ^]^
Mercury ion (Hg^2+^)	Au@Ag core/shell NPs	R6G	0.1 Nm–1 µM	0.1 nM	^[^ ^196^ ^]^
miR‐K12‐5‐5p	Au/Ag/porous GaN	R6G		8.84 × 10^−10^ M	^[^ ^217^ ^]^
miRNA‐21	Au@Ag@4MBA, Ag/TiO_2_ nanowires	4‐MBA	1.0 fM–1.0 nM	0.75 fM	^[^ ^214^ ^]^
miRNA‐21	Au@DTNB@Ag core–shell	DTNB	0–1 nM	0.084 fM	^[^ ^216^ ^]^
miR‐1246, miR‐221, miR‐133a, and miR‐21	Au–Ag NPs	MPBN	‐	0.15 pM	^[^ ^218^ ^]^
miR‐196a‐5p and miR‐31‐5p	Au–AgNSs@4‐ATP and Au–AgNSs@DTNB	4‐ATP, DTNB	‐	1.171 and 2.251 nM in Phosphate buffer and 1.681 and 2.603 nM in human serum for miR‐196a‐5p, and miR‐31‐5p respectively	^[^ ^156^ ^]^
Chloramphenicol (CAP), thiamphenicol (TAP), and florfenicol (FFC)	core–shell Au@Ag NPs	4‐MBA	‐	0.36 ng/ml for CAP, 0.20 ng/ml for TAP, and 0.78 ng/ml for FFC	^[^ ^157^ ^]^
*E. coli* O157:H7	Au@DTNB@AgDTNB	DTNB	‐	6.94 × 10^1^ CFU/ml	^[^ ^155^ ^]^
Methylamphetamine	Au@Ag core–shell nanoparticles	4‐MBA	0.5 ppb–40 ppb	0.16 ppb	^[^ ^229^ ^]^
2,4‐dichlorophenoxyacetic acid (2,4‐D)	HAu@AgNFs@MBA‐antigen	4‐MBA	0.001–100 µg/ml	0.11 ng/ml	^[^ ^230^ ^]^
Melamine	Au/Ag BPHAN array	‐	‐	10^−9^ M	^[^ ^5b^ ^]^
CTC/MCF‐7	Core–shell plasmonic nanorods (PNRs)	4‐mp	‐	20 MCF‐7 cells	^[^ ^143^ ^]^
West Nile Virus (WNV) and non‐structural protein 1 (NS1)	Au/DTNB@Ag/DTNB	DTNB	‐	0.2 × 10^2^ copies/µl for WNV, and 0.1 ng/ml for NS1	^[^ ^231^ ^]^
Influenza A, parainfluenza 1, parainfluenza 3, respiratory syncytial virus, coxiella burnetii, legionella pneumophila, influenza B, parainfluenza 2, adenovirus, chlamydophila pneumoniae, and mycoplasma pneumonia	AgMB@Au and AgNBA@Au NPs	NBA, MB	1 pM–50 nM	0.031 pM, 0.030 pM, 0.038 pM, 0.038 pM, 0.040 pM, 0.039 pM, 0.035 pM, 0.032 pM, 0.040 pM, 0.039 pM, and 0.041 pM, respectively	^[^ ^232^ ^]^
SARS‐CoV‐2 RBD protein (RBD)	Au/Ag nanostructures	4‐NTP	‐	1 pM	^[^ ^117c^ ^]^
E. coli, *S. aureus*, and *P. aeruginosa*	Au@Ag‐GO	4‐MPBA	‐	10^1^ CFU/ml	^[^ ^130^ ^]^
*E. coli*	Au@AgNRs	R6G	‐	10^2^ CFU/ml	^[^ ^131^ ^]^
*Pyocyanin*	Au@Ag NSs	‐	‐	335 pM	^[^ ^135^ ^]^
*S. aureus*, *E. coli*, *S. dysenteriea*, *P. aeruginosa*, and *K. pneumonia*	Au@Ag@PDA	pATP	‐	10 CFU/ml	^[^ ^132^ ^]^
*S. aureus*, *E. coli*, and *L. monocytogenes*	Au@Ag NPs	4‐MBA	‐	10^4^ CFU/ml	^[^ ^137^ ^]^
*E. coli*, *V. anguillarum*, *V. harveyi*, *E. piscicida*, and *P. plecoglossicida*	Au@Ag core–shell	4‐MPY	‐	10^5^ CFU/ml	^[^ ^136^ ^]^

## 5 FUTURE ASPECTS

Scientific research has enabled pharmaceutical industry to cater to the industrial and market requirement by producing nanoscale materials for biological therapeutic, diagnostic, and medicinal applications. There are still many challenges to advancing the efficiency of detection, decreasing the interventions from molecules in the matrices, and photoluminescence from augmenting substrates. SERS technique has a great importance in biological detection; however, it still requires more up‐gradation for practical applications.

The viral detection is still not properly explored in this technique due to lack of factual samples from prevalent cases, which is a very key element in current pandemic situation for accurately diagnosing and treating COVID‐19 virus. In a latest study, a projected approach will have the advantage of making COVID‐19 viral detection time effective with any advanced medication.^[^ ^123^ ^]^ Similarly, Raman technique has a great tendency and potential to be applied for different pathogenic sensing. The contribution of efficient SERS substrates will be a key for this kind of applications. In case of in vivo uses for therapeutic analysis and detection, SERS sensing based on nanoscale materials will have drawbacks due to problems like intense noise signals by the tissues samples, and the diffusion extent of laser‐beam for locating intended spot (cancel cell) in human bio‐system, and a very focused research efforts will have to dedicated to deal with it. The recent breath‐based COVID‐19 detection by conventional gas mass‐spectrometry method has provided an opportunity to comparatively work on the SERS based breath‐based COVID‐19 detection for making it an equally efficient and cost‐effective method. It will also be very encouraging for further exploring the SERS sensing based studies for other respiratory and/or nonrespiratory related diseases.^[^ ^118^ ^]^

In past two decades, the anisotropic type of nanomaterials based on gold and silver, and their bimetallic alloy have made a mark in scientific field. There have been numerous effective and productive efforts for the various preparation methods of these bimetallic alloy nanomaterials. The phenomenon of intense mono‐dispersibility and optimum regulation of structural framework are key factors in establishing new synthetic procedures. The optical features of nanomaterials for practical applications essentially hinge on the basic features of conformational measurements and dimensions. So, the major point in the direction of this research will be the extensive efforts for the large‐scale production of different types of metallic NPs, especially very efficient bimetallic Au–Ag alloy shell for diverse applications.

The anisotropic bimetallic Au–Ag metallic alloy nanomaterials contain exceptionally upgraded and expanded electric field at its honed junctions and curves underneath an extensive array of excitation wave features with reference to UV–vis to near‐IR region. It gives bimetallic core–shell alloy nanoscale materials extreme importance as adaptable for application in plasmonic upgrading materials for methodologies like metal‐enhanced fluorescence (MEF) and SERS.

The potential to sense various biomarkers like aptamers and antibodies instantaneously makes core@shell alloy very critical for many uses in biological‐samples and toxin‐contaminated locations, which holds great potential for modified and customized highly efficient therapeutic diagnosis, treatment, and medicinal products established on POC at the genetic and molecular stages. Label‐free SERS bio‐detection proposes DNA sensing by a single hybridization step, subsequently giving rise to a reduced evaluation period, and a lesser amount of substance consumed. SERS bioimaging utilized by bimetallic Au–Ag core@shell alloy is becoming extraordinarily selective and multiplexing, despite dodging photo‐bleaching, which is frequently faced in fluorescence sensing systems. Moreover, Immunoassay platforms attached to innovative POC strategies have high preference and value for the quick biological testing. This trend of expansions and innovations in nanoscale bimetallic Au–Ag alloy evolution for SERS application will be pivotal for prospective field of adaptive genomic drugs, diagnostic techniques, and treatments. The impressive works like near‐infrared surface‐enhanced Raman spectroscopy NIR‐SERS sensor for ultra‐sensitive LA (lactic acid) fluctuation monitoring in human sweat and diverse impactful characteristics will pave a way for more sensitive and focused efforts of monitoring key vitals in human body which will ultimately improve standard of human health.^[^ ^226^ ^]^

Although, core–shell Au–Ag nanoparticles and their role in SERS have achieved high scale of applicability in various biosensing directions, the research in area of making Au–Ag materials and related techniques useful for large scale clinical diagnostics technology is still not fast enough. The reasons are the utilization of extremely diluted samples in complex matrixes, and the necessity of refined analytical equipment. These problems could be dealt to some extent by employing brighter probes like SERS tags joined with microfluidic structures and utilizing transportable instruments. In the light of previous studies and the excellent efficient exhibition of characteristic properties of core–shell alloys SERS substrates, it is projected that there are central clinical applications of SERS that could be practically realized sooner: (a) In surgical treatments for tumor detection; (b) in endoscopy, colonoscopy, and other ocular fiber‐steered imaging methodologies to picture, and to sense apparent unhealthy tissues within the body; (c) in liquid biopsy. The efficient and early diagnosis of diseases is a primary challenge, especially in low‐ and middle‐income countries. SERS biosensors based on core–shell Au–Ag alloy materials have the potential to be a cost‐effective and helpful substitute to histopathological examination.

The bimetallic alloy‐based biological sensors with reference to SERS techniques have been discussed and thoroughly analyzed here with a prospective revolution of nanoscale gold and silver materials‐based pharmaceutical industry. The future path of this evolution and realization will need more comprehensive work on conceptual as well as practical aspects including computational studies, basic characteristic profiling, exploration of more innovation in nanoscale materials, especially the thoroughly studied bimetallic alloy from other similar research works. The biosensing and bio‐detection of several molecular constituents in intricate combinations is very effectively performed with help of bimetallic core–shell alloys due to the untiring work on these potentially effective and efficient materials.

## CONFLICT OF INTEREST

Aiguo Wu is a member of the *Exploration* editorial board. The other authors declare no conflict of interest.
